# Supplementary material for: Screening for Hypertension in adolescents living with HIV: Protocol for a cluster randomized trial to improve guideline adherence
Source: PLoS One. 2024 May 3;19(5):e0302016. doi: 10.1371/journal.pone.0302016 (PMC11068165; doi:10.1371/journal.pone.0302016)
Supplement: S1 Protocol — (DOCX) [file pone.0302016.s002.docx]

**SCHOOL OF PUBLIC HEALTH**

**COLLEGE OF HEALTH SCIENCES**

**UNIVERSITY OF GHANA LEGON**

**Using a Theory-Based Intervention to Improve Diagnosis and Management of Hypertension among Adolescents Living with HIV; A Cluster-randomized study**

**BY**

**RAPHAEL ADU-GYAMFI**

**(10161991)**

**A PROPOSAL SUBMITTED TO THE SCHOOL OF PUBLIC HEALTH, UNIVERSITY OF GHANA, LEGON, IN PARTIAL FULFILMENT FOR THE AWARD OF THE DOCTOR OF PUBLIC HEALTH (PhD) DEGREE IN PUBLIC HEALTH**

**Table of Contents**

[**List of tables** 4](#_Toc101391081)

[**List of figures** 4](#_Toc101391082)

[**List of Abbreviations** 5](#_Toc101391083)

[**Study Summary** 5](#_Toc101391084)

[**CHAPTER 1: INTRODUCTION** 7](#_Toc101391085)

[**1.1 Hypertension in adults and adolescents** 7](#_Toc101391086)

[**1.2 HIV, Cardiovascular diseases (CVD) and hypertension** 7](#_Toc101391087)

[**1.3 Guidelines for screening and management of hypertension among children and adolescents.** 9](#_Toc101391088)

[**1.4 Adherence to clinical practice guidelines** 9](#_Toc101391089)

[**1.5 Theory of planned behaviour** 10](#_Toc101391090)

[**1.6 Problem statement** 10](#_Toc101391091)

[**1.7 Justification** 12](#_Toc101391092)

[**1.8 Research questions** 12](#_Toc101391093)

[**1.9 Aim and objectives** 13](#_Toc101391094)

[**1.10 Study hypothesis** 14](#_Toc101391095)

[**CHAPTER 2: LITERATURE REVIEW** 15](#_Toc101391096)

[**2.1 Cardiovascular Diseases (CVD)** 15](#_Toc101391097)

[**2.2 Epidemiology of CVDs** 15](#_Toc101391098)

[**2.3 Importance of CVDs** 16](#_Toc101391099)

[**2.4 Hypertension** 17](#_Toc101391100)

[**2.4.1 Adolescent hypertension** 18](#_Toc101391101)

[**2.4.2 Effect of uncontrolled hypertension in children and adolescents** 19](#_Toc101391102)

[2**.5 HIV Cardiovascular disease and hypertension** 19](#_Toc101391103)

[**2.5.1 The Human Immunodeficiency Virus (HIV) Infection** 19](#_Toc101391104)

[**2.5.2 HIV and CVDs** 20](#_Toc101391105)

[**2.5.3 HIV and hypertension.** 21](#_Toc101391106)

[**2.5.4 Adolescents living with HIV and hypertension.** 22](#_Toc101391107)

[**2.6 Clinical Practice Guidelines** 23](#_Toc101391108)

[**2.6.1 Guidelines for screening and management of hypertension among children and adolescents.** 23](#_Toc101391109)

[**2.6.2 Screening for hypertension** 23](#_Toc101391110)

[**2.6.3 Diagnosis of hypertension** 23](#_Toc101391111)

[**2.6.4 Investigating the aetiology of hypertension** 26](#_Toc101391112)

[**2.6.5 First-line management of hypertension** 27](#_Toc101391113)

[**2.6.7 Referral** 27](#_Toc101391114)

[**2.6.8 Guidelines for screening and management of hypertension among children and adolescents in Ghana** 28](#_Toc101391115)

[**2.7 Adherence to clinical practice guidelines** 28](#_Toc101391116)

[**2.7.1 Adherence to Guidelines for screening and management of hypertension among children and adolescents** 29](#_Toc101391117)

[**2.7.2 Factors affecting HCW adherence to guidelines** 29](#_Toc101391118)

[**2.8 Theoretical determinants of healthcare worker behaviour towards clinical practice guidelines** 31](#_Toc101391119)

[**2.8.1 The Theory of Planned Behavior (TPB)** 31](#_Toc101391120)

[**2.8.2 Previous guidelines adherence research among healthcare workers using TPB** 33](#_Toc101391121)

[**2.9 Interventions to improve healthcare worker adherence to clinical practice guidelines** 34](#_Toc101391122)

[**2.9.1 Continuous Professional Development Programmes** 35](#_Toc101391123)

[**2.9.2 Community-based interventions** 35](#_Toc101391124)

[**2.9.3 Practice-based interventions** 35](#_Toc101391125)

[**2.10 Gaps in the literature** 36](#_Toc101391126)

[**2.11 Conceptual Framework** 37](#_Toc101391127)

[**CHAPTER 3: METHODOLOGY** 40](#_Toc101391128)

[**3.1 Introduction** 40](#_Toc101391129)

[**3.2 Non-experimental component** 40](#_Toc101391130)

[**3.2.1 Behaviour of Interest** 40](#_Toc101391131)

[**3.2.2 Study design** 40](#_Toc101391132)

[**3.2.6 TPB-Based Survey among Health Workers** 44](#_Toc101391133)

[**3.3 Experimental Component** 47](#_Toc101391134)

[**3.3.1 Study Design** 47](#_Toc101391135)

[**3.3.2 Study Settings** 47](#_Toc101391136)

[**3.3.3 Participants** 47](#_Toc101391137)

[**3.3.4 Recruitment** 48](#_Toc101391138)

[**3.3.5 Sample size** 49](#_Toc101391139)

[**3.3.6 Randomization** 50](#_Toc101391140)

[**3.3.7 Intervention** 51](#_Toc101391141)

[**3.3.8 Comparator** 51](#_Toc101391142)

[**3.3.9 Outcomes** 53](#_Toc101391143)

[**3.4 Data Management** 56](#_Toc101391144)

[**3.4.1 Data Quality Control** 56](#_Toc101391145)

[**3.4.2 Data Analysis** 56](#_Toc101391146)

[**3.5 Expected Outcome** 58](#_Toc101391147)

[**3.6 Ethical Consideration** 58](#_Toc101391148)

[**3.6.1 Voluntary Participation** 58](#_Toc101391149)

[**3.6.2 Privacy and Confidentiality** 59](#_Toc101391150)

[**3.6.3 Risks** 59](#_Toc101391151)

[**3.6.4 Compensation** 59](#_Toc101391152)

[**3.6.5 Declaration of Conflict Of Interest** 59](#_Toc101391153)

[**3.7 Funding** 59](#_Toc101391154)

[**3.8 Protocol Amendments** 59](#_Toc101391155)

[**4.0 References** 65](#_Toc101391156)

[**5.0 Appendix** 88](#_Toc101391157)

[**Appendix I: Participant information sheet and consent form(1)** 88](#_Toc101391158)

[**Participant Agreement for Elicitation Study** 91](#_Toc101391159)

[**Appendix II: Questions for elicitation study** 92](#_Toc101391160)

[**Appendix III: Participant information sheet and consent form (2)** 93](#_Toc101391161)

[**Participant Agreement for Theory of planned behavior-based questionnaire** 96](#_Toc101391162)

[**Appendix IV: Parental information sheet and consent form(3)** 97](#_Toc101391163)

[**VOLUNTEER AGREEMENT** 100](#_Toc101391164)

[**Appendix V: Adolescent information sheet and assent form (4)** 101](#_Toc101391165)

[**VOLUNTEER AGREEMENT** 104](#_Toc101391166)

# **List of tables**

[**Table 1 Application of Theory of Planned behavior to intervention design and measurement** 51](#_Toc99624905)

[**Table 2 Study Timelines** 60](#_Toc99624906)

[**Table 3 Study Budget** 61](#_Toc99624907)

# **List of figures**

**Figure 1 (A) Blood pressure (BP) categories definitions and management algorithm (B) Minimum systolic and diastolic BP that warrants further investigation for girls and boys based on age(Flynn et al., 2017)** 23

**Figure 2 Theory of Planned Behaviour(Ajzen, 1991)** 31

**Figure 3 Conceptual Framework** 36

**Figure 4 Flow chart of Cluster randomized Study Procedures** 55

# **List of Abbreviations**

**AIDS** Acquired Immune Deficiency Syndrome

**ADLHIV** Adolescents living with HIV

**ANOVA** Analysis of Variance

**ART** Antiretroviral Therapy

**BP** Blood Pressure

**CAVI** Cardio-Ankle Vascular Index

**CPD** Continuous Professional Development

**CPG** Clinical Practice Guidelines

**CVD** Cardiovascular Disease

**DBP** Diastolic Blood Pressure

**GHS** Ghana Health Service

**HCW** Healthcare worker

**HIV** Human Immunodeficiency Virus

**NHBPEP** National High Blood Pressure Education Program

**PLHIV** Persons living with HIV

**SBP** Systolic Blood pressure

**STG** Standard Treatment Guidelines

**TPB** Theory of Planned Behavior

**TRA** Theory of Reasoned Action

**WHO** World Health Organization

# **Study Summary**

**Background:** Recent studies have shown a relatively higher incidence of cardiovascular diseases and related deaths among persons living with HIV than uninfected persons. The adverse effects of some antiretroviral medication, chronic vascular inflammation, and immune activation from persistent HIV replication leading to arterial stiffness contribute to this. The duration of exposure to the virus plays a vital role in the pathogenesis, putting perinatally infected children and adolescents at higher risk than behaviorally-infected individuals. This, therefore, supports the calls for increased surveillance of NCDs, especially hypertension among them.

**Aim and objectives:** The overall aim of the study is to improve the adherence of healthcare workers to the guidelines for screening and management of hypertension among adolescents living with HIV(ADLHIV) at selected ART sites in the Greater Accra Region. The objectives are to

1. Determine the factors influencing health care workers' adherence to the guidelines for screening and management of hypertension among ADLHIV.
2. Assess the effect of a Theory of Planned Behavior-based intervention on healthcare workers' adherence to guidelines for screening & management of hypertension among ADLHIV.
3. Assess the effect of a Theory of Planned Behavior-based intervention on the arterial stiffness and blood pressure of ADLHIV.

**Methodology:** The study will be conducted among 24 antiretroviral therapy sites in the Greater Accra Region. An elicitation study will be conducted to develop a theory of planned behavior-based questionnaire. This will be used in a survey to determine the factors influencing healthcare workers' intention to check the blood pressure of adolescents living with HIV. A cluster-randomized study will be conducted to assess the effect of a theory of planned behavior-based intervention on healthcare worker adherence to the guidelines as well as the blood pressure and arterial stiffness of adolescents living with HIV after a six-month follow-up period.

# **CHAPTER 1: INTRODUCTION**

## **1.1 Hypertension in adults and adolescents**

Hypertension is the leading modifiable risk factor for cardiovascular disease (CVD)-related disability and mortality globally. Each 10 mmHg increase in systolic blood pressure (BP) is associated with a 45% higher risk of ischemic heart disease and about a 65% higher risk of ischaemic or haemorrhagic stroke in those aged 55–64 years(Singh et al., 2013). In 2019, it was estimated to have accounted for 10.8 million deaths (19.2% of total deaths) and 235 million disability-adjusted life years (9.3% of total disability-adjusted life years) worldwide(Abbafati et al., 2020; Mills et al., 2020).

Although thought to be an adult disease, arterial hypertension is established early in life, with a rising prevalence alongside the obesity pandemic. Its prevalence has been reported to range from 0·2–24·8% among adolescents in Sub Saharan Africa(Noubiap et al., 2017) and 9.1% among adolescents in the Ashanti Region of Ghana(Noubiap et al., 2017; Sekyere & Abena, 2018). Elevated BPs in childhood and adolescence predict adult hypertension and increase the risk for cardiovascular morbidity and mortality in adulthood(Oh & Hong, 2019). Children and adolescents with a single BP measurement >90th percentile for the age, sex, and height are 2.4 times more likely to have an adult BP >90th percentile. Early detection, prevention and treatment of adolescent elevated BP could eliminate 10% of adult elevated BPs (Bao et al., 1995a; Kelly et al., 2015; Lauer et al., 1993). In addition to poor dietary and physical activity behaviours, one emerging risk factor for developing hypertension is HIV infection (Benzekri et al., 2018; Kwarisiima et al., 2016).

## **1.2 HIV, Cardiovascular diseases (CVD) and hypertension**

Although AIDS-related deaths have markedly reduced with increased access to antiretroviral care, their CVD-related morbidities are rising(Alonso et al., 2019). Compared to uninfected persons, they experience a higher incidence(per 1000 person-years) of CVD-related admissions( 10.8 vs 5.9), myocardial infarction( 1.4 vs 1.1), heart failure( 3.5 vs 1.1), stroke( 1.4 vs 0.5) and atrial fibrillation( 3.5 vs 2.9) (Alonso et al., 2019). Contributing to this is the higher prevalence of hypertension amongst persons living with HIV(PLHIV)(Bigna et al., 2020; K. Davis et al., 2021; Gebrie, 2020).

A recent meta-analysis of data from around the globe demonstrated that 35% of all HIV-infected adults on ART have hypertension, compared with an estimated 30% of HIV-uninfected adults(Xu et al., 2017). More than half of antiretroviral(ARV)-experienced individuals over 50 years are estimated to have hypertension(Fahme et al., 2018; Mbuthia et al., 2021; Pierre et al., 2019). In addition to the traditional modifiable and non-modifiable risk factors for hypertension, ARV- and HIV-associated microbial translocation, chronic inflammation, immune reconstitution, dyslipidemia, renal disease, and neuroendocrine response lead to endothelial dysfunction, accelerated vascular ageing and arterial stiffness(Fahme et al., 2018).

With a reported incidence(per 1000) of 10- 16 per month after ART initiation, the duration of HIV infection is a significant determinant in the development of hypertension, causing perinatally- and behaviorally- infected adolescents to have higher odds of being hypertensive(Mulugeta et al., 2021; Rodríguez-Arbolí et al., 2017). Adolescents living with HIV(ADLHIV) have been shown to have a higher prevalence of hypertension compared to their uninfected age and sex-matched peers(Chatterton-Kirchmeier et al., 2015; Oh & Hong, 2019). Therefore, the Expert Panel on Integrated Guidelines for Cardiovascular Health and Risk Reduction in Children and Adolescents classified HIV infected children and adolescents as being at moderate risk of CVD, especially hypertension, and called for increased surveillance (De Jesus, 2011b).

Several clinical practice guidelines have been developed for service providers to guide hypertension surveillance among children and adolescents. The most referenced is the Fourth Report by the National High Blood Pressure Education Program (NHBPEP) Working Group on Children and Adolescents(Falkner & Daniels, 2004), which informed the recommendations in Ghana’s Standard Treatment Guidelines(GHS, 2017).

## **1.3 Guidelines for screening and management of hypertension among children and adolescents.**

The guideline defines hypertension in children and adolescents as systolic BP (SBP) or diastolic BP (DBP), which is, on repeated measurement, at or above the 95th percentile for age, sex and height. BP between the 90th and 95th percentile in childhood and adolescence is designated “prehypertensive”. It also provides reference ranges to guide the classification of BP in children and a detailed follow-up plan based on the classification given. In addition, it captures recommendations on the frequency of screening, diagnosis, investigation and management of hypertension among them(Falkner & Daniels, 2004).

## **1.4 Adherence to clinical practice guidelines**

Despite the availability of these guidelines and their adaptations by countries and professional societies, adherence to it by healthcare workers has been generally poor(De Jesus, 2011a; Hagan et al., 2007; Lurbe et al., 2019). There are reports of unchecked blood pressures during two-thirds of adolescent clinic visits(Shapiro et al., 2012), over 80% of hypertensive adolescents not being recognized by healthcare workers (Brady et al., 2010) and about 75% of hypertensive adolescents not being appropriately classified and diagnosed(Hansen et al., 2007). Patient-, provider- and clinic-related factors have been identified as contributing to non-adherence to the clinical practice guidelines(Brady et al., 2010).

Educational, community-based, practice-based and multi-component strategies have been employed to help improve healthcare worker adherence, with differential outcomes(Davis & Taylor-Vaisey, 1997). However, a systematic review by Davis and Taylor recommends interventions targeting predictors of provider behaviour towards these guidelines(Davis & Taylor-Vaisey, 1997). One model which has guided the exploration of healthcare worker intentions to adhere to various guidelines is the Theory of Planned Behavior(TPB)(Ajzen, 1991).

## **1.5 Theory of planned behaviour**

The theory postulates that the likelihood of individuals engaging in a behaviour (e.g. checking the BPs of adolescents) correlates with the strength of their intention to engage in the behaviour. A behavioural intention represents the person’s commitment to act and is itself the outcome of

- the person’s attitudes toward the behaviour (attitude towards adolescent BP screening),
- the person’s perception of subjective group norms concerning the behaviour (subjective norms influencing adolescent BP screening), and
- the extent to which the person perceives him- or herself to have control concerning the behaviour (perceived behavioural control to check BPs of adolescents).

It also states that perceived behavioural control can directly influence behaviour without affecting intentions to perform the behaviour (Fishbein, 2012).

## **1.6 Problem statement**

Ghana is currently in epidemiological transition and is progressing rapidly with urbanization (Agyei-Mensah & Aikins, 2010); stroke and ischemic heart disease rose from being the seventh and ninth leading causes of death respectively in 2000 to take the third and fourth positions in 2019(WHO, 2020b). Contributing to this is the double burden of infectious and non-communicable diseases that increase CVD risk, especially hypertension(Agyei-Mensah & Aikins, 2010; Bosu & Bosu, 2021a).

Hypertension is among the leading causes of morbidity and mortality in Ghana(GHS, 2018; Nuamah et al., 2017), and the outpatient burden has been increasing. In the Greater Accra Region, new cases increased 3.8-fold in five years, from 35,855 in 2006 to 138,040 in 2010(Opare et al., 2013). It was the third leading cause of admissions and the leading cause of death, accounting for 4.7% of the total admissions and 15.3% of the total deaths in Ghana in 2017(GHS, 2018). It is the main determinant of stroke in the country, with a population attributable risk of about 91%(Owolabi et al., 2018). However, approximately two-thirds of hypertensives in the country are undiagnosed(Bosu & Bosu, 2021b).

Adding to the hypertension burden in the country is the increasing HIV burden. With a pooled prevalence of 30.3% (95% CI 26.1–34.8%) among the general population(Atibila et al., 2021) and  41.3% [95% CI, 35.2-47.3] among adult clients on antiretroviral therapy(ART) (Nartey, 2021), HIV is a significant contributor to the hypertension caseload in the country and thus needs to be given much attention.

Although Ghana’s sixth standard treatment guidelines provide directions on diagnosing and managing hypertension among children and adolescents(Andy, 2010), healthcare workers' level adherence to this guideline is unknown. This information is crucial because, in addition to the increase in the prevalence of known traditional risk factors, adolescents in Ghana generally have a relatively higher risk of hypertension due to in utero exposure to malaria(Ayoola et al., 2014; Bedu-Addo et al., 2017; Etyang et al., 2019). .

Concerning adolescents living with HIV, over 80% of the 23,000 in the country had the infection perinatally(UNAIDS, 2021a) and thus have more prolonged exposure to the virus and metabolic effects of the antiretrovirals. This makes them more likely to develop hypertension, especially those exposed to lopinavir- and dolutegravir-based regimen(WHO, 2021a).

The call by WHO to integrate hypertension and diabetes management into HIV services and the support for task shifting in HIV and hypertension care in the country (BeLue et al., 2009; P. Patel et al., 2018; UNAIDS, 2008; WHO, 2020a) provide a healthy policy environment for reducing the future burden of hypertension among these adolescents and for protecting those already hypertensive from the negative impact of COVID-19 through early diagnosis and appropriate management.

## **1.7 Justification**

The Greater Accra Region has over a quarter(28%) of the adolescents living with HIV in the country(Ghana AIDS Commission, 2021b). The ART-providing facilities in the Region will therefore be an excellent place to undertake an assessment of the level of healthcare worker adherence to the guidelines, and to identify contextual factors contributing to differential adherence among them, based on the theory of planned behaviour. Findings from the study will help develop an evidence-based intervention to improve healthcare workers' adherence to clinical practice guidelines and provide a framework to guide the effective dissemination of these guidelines in the country.

## **1.8 Research questions**

To help reduce the anticipated hypertension-related morbidities and mortalities among adolescents living with HIV, adherence to the guidelines for screening and management of hypertension among children and adolescents is critical at facilities providing ART services. This study, therefore, seeks to answer the following questions concerning ART sites in the Greater Accra Region:

1. Do service providers at ART sites adhere to the guidelines for screening and management of hypertension among adolescents?
2. What factors influence adherence to guidelines for screening and management of hypertension among adolescents?
3. Will a theory of planned behaviour-based intervention for service providers at ART sites improve adherence to guidelines for screening and management of hypertension among ADLHIV?
4. Will a theory of planned behavior-based intervention for service providers at ART sites improve the arterial stiffness of adolescents living with HIV who are found hypertensive?

## **1.9 Aim and objectives**

The overall aim is to improve the adherence to the CPG for diagnosis & management of hypertension among ADLHIV at selected ART sites in the Greater Accra Region.

The objectives are to

- 1. Determine the factors influencing adherence to the CPG for screening & management of hypertension among ADLHIV
  2. Assess the effect of a Theory of Planned Behavior-based intervention on HCW adherence to guidelines for screening & management of hypertension among ADLHIV
  3. Assess the effect of a Theory of Planned Behavior-based intervention on the arterial stiffness of ADLHIV found to have high BP.

## **1.10 Study hypothesis**

The hypotheses that will be tested in the study are as follows:

1. There is no difference in the proportion of adolescent ART visits that had BPs checked between facilities exposed to the intervention and unexposed facilities.
2. There is no change in arterial stiffness parameters of hypertensive adolescents living with HIV after six months of exposure to the TPB- based intervention.

# **CHAPTER 2: LITERATURE REVIEW**

## **2.1 Cardiovascular Diseases (CVD)**

Cardiovascular Diseases (CVD) affect the heart and blood vessels, leading predominantly to coronary artery disease, stroke, and peripheral arterial disease. They have assumed public health significance globally(Liu, 2014). In 2011, the United Nations (UN) formally recognized non-communicable diseases, including CVDs, as a major concern for global health and set out an ambitious plan to dramatically reduce their adverse effects in all regions. The sixth World Health Assembly in 2013 and the Sustainable Development Goals provided guidance for UN member states to achieve a one-third reduction in premature mortality due to these diseases by 2030(Alwan et al., 2011; United Nations, 2021).

## **2.2 Epidemiology of CVDs**

The Global Burden of Diseases estimates report (2019) showed that the number of persons living with CVDs almost doubled from 270 million in 1990 to 523.2 million in 2019 globally. An estimated 18.6 million CVD-related deaths occurred in 2019, contributing to about a third (32.8%) of all deaths globally and leading to a total of 393 million disability-adjusted life years lost. Over 85% of these CVD-related deaths resulted from ischemic heart disease and stroke, two diseases that maintained their lead as the major causes of death globally since 2000(NCD Allicance, 2019; Schettler & Brisse, 2017; WHO, 2019).

More than three-quarters of the CVD-related deaths took place in low- and middle-income countries, and out of the 17 million premature deaths (under the age of 70) due to non-communicable diseases in 2019, 82% occurred in these countries, with 37% caused by CVDs(World Health Organization, 2020). Whilst the CVD caseload doubled globally from 1990 to 2019, the number of persons living with these conditions almost tripled over the same period in Ghana. The Institute for Health Metrics and Evaluation estimated that CVDs contributed to 19% of all deaths in the country in 2019, and this proportion has also seen a sturdy rise since 2000(IHME, 2019).

## **2.3 Importance of CVDs**

#### **Economic Importance**

In addition to the staggering toll they take on human lives and health, CVDs wreak great havoc on countries' economies. They contributed to 55% of direct health costs and a 45% reduction in productivity globally in 2018(World Heart Federation, 2019). In the United States, CVDs were the most costly diseases with a price tag of $555 billion in 2016. Their economic burden is projected to worsen and be in trillions of dollars by 2035(RTI International, 2016). A systematic review by Gheorghe and colleagues on the economic burden of CVDs in Lower-Middle Income Countries(LMIC) suggested that the average costs per episode for hypertension and other generic CVDs ranged between $500 and $1500, whilst that for coronary heart disease (CHD) and stroke were in excess of $5000 per episode. They found the average monthly cost for hypertension treatment to be $22 whilst that for stroke and CHD ranged between $300 and $1000. In Summary they noted that these costs exceeded the total health expenditure per capita in most LMICs(Gheorghe et al., 2018). By 2030 the total global cost of CVD is set to rise from approximately US$863 billion in 2010 to a staggering US$1,044 billion(World Heart Federation, 2019).

#### **Comorbid effect of CVDs on COVID-19.**

The ongoing COVID -19 pandemic, caused by infection with the Severe Acute Respiratory Syndrome Coronavirus 2 (SARS-CoV-2), has further shown the clinical importance of CVDs (Chung et al., 2021). Over 40% of confirmed COVID-19 patients that required hospitalization reportedly had a history of cardiovascular diseases and their risk factors(Bax et al., 2020; Nishiga et al., 2020; Ogah et al., 2021; S. Shi et al., 2020). Several authors observed that a large proportion of deaths from infected patients could be attributed to cardiovascular diseases, including acute myocardial infarction, arrhythmias and heart failure(Bansal, 2020; Chatterjee & Cheng, 2020; Yi et al., 2021). A meta-analysis by Bae reported patients with CVDs to have a higher risk of developing the severe disease (44.4% vs 23.3%, OR 3.15, 95% CI 2.34 to 4.25) and death (35.7% vs 17.6%, OR 3.23, 95% CI 2.28 to 4.57) than those without CVDs(Bae et al., 2021). CVDs, therefore, amplified the lethal effect of the virus.

## **2.4 Hypertension**

Hypertension, defined as persistently high blood pressure (systolic BP ≥140 mmHg or the diastolic BP ≥90 mmHg or both), is the leading modifiable risk factor for CVD-related disability and mortality globally. In 2019, it was estimated to have accounted for 10.8 million deaths (19.2% of total deaths) and 235 million disability-adjusted life years globally (9.3% of total disability-adjusted life years)(Abbafati et al., 2020; Mills et al., 2020).

A meta-analysis by Lewington and colleagues found a doubling of the risk of ischemic heart disease and stroke with every 20 mmHg and 10 mmHg increase in SBP and DBP, respectively, starting from as low as 115 mmHg for SBP and 75 mmHg for DBP(Lewington et al., 2002). An Asian Pacific Cohort Studies Collaboration found similar associations, with each 10 mmHg increase in SBP found to be associated with a 45% higher risk of ischemic heart disease and about a 65% higher risk of ischemic or hemorrhagic stroke in those aged 55–64 years(Singh et al., 2013) Blood pressure rises with age and is higher in obese and overweight individuals (Kotchen, 2011). However, the onset of hypertension predates adulthood(Sekyere & Abena, 2018).

### **2.4.1 Adolescent hypertension**

Although thought to be an adult disease, arterial hypertension is established early in life. Early-onset hypertension is a predictor of adult hypertension and increases the risk of cardiovascular morbidity and mortality in adulthood(Oh & Hong, 2019). Studies have demonstrated that children with a single BP measurement >90th percentile are 2.4 times more likely to have an adult BP >90th percentile, and preventing pediatric elevated BP could eliminate 10% of adult elevated BP(Bao et al., 1995a; Kelly et al., 2015; Lauer et al., 1993). These and other findings have led to a paradigm shift from primary adult prevention to primordial prevention of hypertension in childhood(De Jesus, 2011b; Lloyd-Jones et al., 2010). Early recognition and management of children at high risk and those with hypertension are essential to decrease the substantial adult CVD burden(Flynn et al., 2017).

Song and colleagues reported a global pooled prevalence rates of 4.0% (95% confidence interval [CI]: 3.3%-4.8%) for hypertension and 9.7% (95% CI: 7.3%-12.4%) for prehypertension among persons 10-17 years(Song et al., 2019). Data from a population of over 54,000 children and adolescents aged 2-19 years across Africa gave a pooled prevalence of 5.5% (95% CI: 4.2-6.9) for elevated blood pressure (systolic or diastolic blood pressure ≥ 95^th^ percentile) and 12.7% (95% CI: 2.1-30.4) for slightly elevated blood pressure (systolic or diastolic blood pressure ≥ 90^th^ percentile and < 95^th^ percentile)(Noubiap et al., 2017). About 9.1% of adolescents in the Ashanti Region of Ghana were also found hypertensive (Sekyere & Abena, 2018). In all populations, the prevalence of elevated blood pressure was strongly associated with body mass index (BMI), being six times higher in obese children compared to those with normal weight(30.8% vs 5.5%; p<0.0001)(Noubiap, 2020).

### **2.4.2 Effect of uncontrolled hypertension in children and adolescents**

Hypertension in children is increasingly recognized as a serious public health problem, not only because of its rising prevalence with obesity but the growing evidence that elevated blood pressure early in life has detrimental lifelong cardiovascular effects(Bao et al., 1995b; Falkner & Lurbe, 2020; Theodore et al., 2015). In a recent systematic review and meta-analysis, elevated blood pressure in childhood or adolescence was significantly associated, in adulthood, with indicators of end-organ damage such as high pulse wave velocity or arterial stiffness (pooled odds ratio [OR] 1.83, 95% CI: 1.39-2.40), high carotid intima-media thickness (OR 1.60, 95% CI: 1.29-2.00) and left ventricular hypertrophy (OR 1.40, 95% CI: 1.20-1.64)(Yang et al., 2020). In youth, elevated blood pressure was also linked with cardiovascular disease and mortality in adulthood(Yang et al., 2020). After 20 years of following up a cohort of 8,720 adolescents found hypertensive during a medical examination, they were observed to have a relatively higher mortality risk due to CVD compared to the normotensive adolescents( Hazard ratio=1.5), with an exceptionally high risk for stroke mortality(Hazard ratio= 3.0)(Leiba et al., 2015).

Hypertension at any age is therefore associated with adverse cardiovascular outcomes, supporting the call by the World Health Assembly in 2013 for a 25% decrease in the prevalence of raised BP from its 2010 level by 2025 through risk factor reduction(Zhou et al., 2021). In addition to poor dietary and physical activity behaviours, smoking and alcohol abuse, one emerging risk factor for the development of hypertension is infection with the Human Immunodeficiency Virus (HIV) (Benzekri et al., 2018; Kwarisiima et al., 2016).

## 2**.5 HIV Cardiovascular disease and hypertension**

### **2.5.1 The Human Immunodeficiency Virus (HIV) Infection**

According to the World Health Organization, approximately 37.7 million people were living with HIV globally as of December 2020, over two-thirds of whom were in the WHO African Region and about 346,000 in Ghana(Ghana AIDS Commission, 2021a; WHO, 2021b). Adolescents and young persons form a growing proportion of people living with HIV(PLHIV) worldwide, and a focus on them is required to end AIDS and its sequelae as public health threats(UNICEF, 2020). In 2020, 1,750,000 adolescents were estimated to be living with HIV globally, with 150,000 of them being newly infected. The majority were in sub-Saharan Africa and about 23,000 in Ghana(Ghana AIDS Commission, 2021b; UNAIDS, 2021b).

Although there is no cure for HIV, increasing access to antiretroviral therapy, including treatment for opportunistic infections, has transformed the infection into a chronic disease. As a consequence, it is estimated that by 2030, the median age of PLHIV would increase from 43∙9 years in 2010 to 56∙5 years in 2030, by which time, 78% of them would have age-associated CVDs (Hsue & Waters, 2018; Smit et al., 2015).

### **2.5.2 HIV and CVDs**

Recent evidence has shown a relatively higher incidence of CVDs and CVD-related deaths among PLHIV than uninfected persons. A review by Alonso and colleagues reported that compared to uninfected persons, HIV positive persons had a higher incidence(per 1000 person-years) of CVD-related admissions( 10.8 vs 5.9), myocardial infarction( 1.4 vs 1.1), heart failure( 3.5 vs 1.1), stroke( 1.4 vs 0.5) and atrial fibrillation( 3.5 vs 2.9)(Alonso et al., 2019). Contributing to this is the relatively higher prevalence of CVD risk factors, particularly hypertension, amongst them. Appiah and colleagues, in a survey in Ghana, found a higher prevalence of hypertension ( 29% vs 15%), dyslipidemia(9% vs 5%) and diabetes(5% vs 0.6%) among persons living with HIV, compared to uninfected volunteers(Appiah et al., 2019).

### **2.5.3 HIV and hypertension.**

Hypertension is an emerging challenge among persons living with HIV (PLHIV). (Fahme et al., 2018; Peck et al., 2014; Xu et al., 2017). HIV-infected adults on treatment have a higher hypertension prevalence than uninfected individuals(Peck et al., 2014). A global meta-analysis suggests that 35% of all HIV-infected adults on ART have hypertension, compared with an estimated 30% of HIV-uninfected adults(Xu et al., 2017). More than half of ART-experienced individuals over 50 years are estimated to have hypertension(Fahme et al., 2018; Mbuthia et al., 2021; Pierre et al., 2019).

#### **Pathophysiology**


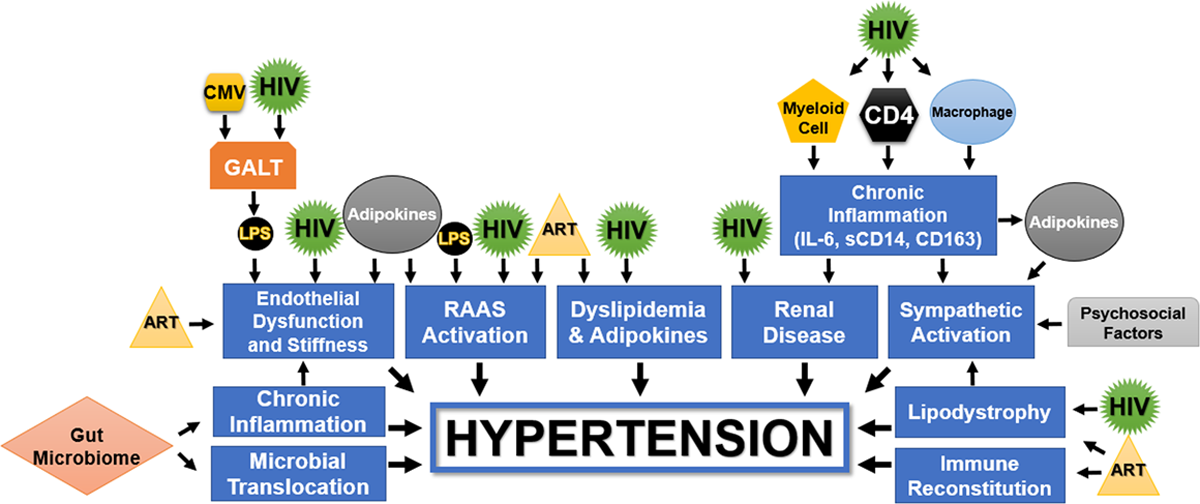


Figure 1Schematic representation of HIV-related mechanisms of hypertension(Fahme et al., 2018)

**Schematic representation of HIV-related mechanisms of hypertension(Fahme et al., 2018)**

Although older age, obesity, male gender family history of hypertension and level of physical activity play a role in the genesis of hypertension amongst PLHIV (Fiseha et al., 2019), contributing to their higher incidence and prevalence are the effects of ARV- and HIV-associated microbial translocation, chronic inflammation, immune reconstitution, dyslipidemia, renal disease and neuroendocrine response. These, directly and indirectly, lead to endothelial dysfunction, accelerated vascular ageing and arterial stiffness(figure 1) (Fahme et al., 2018). With a reported incidence (per 1000) of 10- 16 per month after ART initiation, the duration of HIV infection and treatment plays a significant role in the development of hypertension, putting perinatally- and behaviorally- infected adolescents at higher risk (Mulugeta et al., 2021; Rodríguez-Arbolí et al., 2017).

### **2.5.4 Adolescents living with HIV and hypertension.**

Like adults, HIV positive adolescents are at a relatively higher risk of hypertension than their uninfected age and sex-matched peers. Among adolescents living with HIV(ADLHIV) in Lagos, Adeloye et al. had a hypertension prevalence of 10.5%, with older age [odds ratio (OR) 1.557, 95% confidence interval (CI) 0.344–7.040], waist circumference (OR 6.435, 95% CI 1.396–29.666), duration on ART more than eight years (OR 1.308, 95% CI 0.332–5.153), and dyslipidemia (OR 2.942, 95% CI 0.726–11.914) predicting the risk of hypertension among them. (Adeloye et al., 2015). Ryscavage and colleagues found that perinatally infected adolescents have higher odds (3.4 (95% CI 1.48–7.66)) of developing hypertension than HIV-uninfected peers. After controlling for sex, race, and family history of hypertension, they still had 4.7 times the odds of being hypertensive(Ryscavage et al., 2019). These and other findings contributed to recommendations by the Expert Panel on Integrated Guidelines for Cardiovascular Health and Risk Reduction in Children and Adolescents to classify HIV infected children and adolescents as being at moderate risk of CVD, especially hypertension and calling for increased surveillance among them(De Jesus, 2011b).

To guide and standardize the surveillance for hypertension among children and adolescents, several clinical practice guidelines have been developed for service providers.

## **2.6 Clinical Practice Guidelines**

Field and Lohr have described clinical practice guidelines (CPG) as "systematically developed statements to assist practitioner decisions about appropriate health care for specific clinical circumstances" (Field & Lohr, 1990). Guidelines enhance patient care quality and promote patient safety through evidence-based practice(Dahm et al., 2009; Harrison et al., 2010; Horvath et al., 2010). They are also used to standardize clinical interventions for which health professionals may be held accountable(G. P. Browman, 2000; George P. Browman, 2005; Carlsen et al., 2007; Carlsen & Norheim, 2008).

### **2.6.1 Guidelines for screening and management of hypertension among children and adolescents.**

To help identify, prevent and appropriately manage hypertension in children and adolescents, several guidelines have been developed and updated, with adaptations done by various countries and professional bodies. The most commonly referenced is the Fourth Report by the National High Blood Pressure Education Program (NHBPEP) Working Group on Children and Adolescents(Falkner & Daniels, 2004). The report provides specific consensus and evidence-based statements on screening, diagnosis, investigation and management of hypertension among children and adolescents.

### **2.6.2 Screening for hypertension**

The guideline recommends annual screening for children and adolescents, beginning at three (3) years. However, more frequent screening was recommended for those with specific hypertension risks such as obesity, diabetes, renal disease, and those on treatment with drugs known to increase blood pressure(such as antiretroviral)(Saini et al., 2021).

### **2.6.3 Diagnosis of hypertension**

The guideline defined hypertension in children and adolescents as systolic BP (SBP) or diastolic BP (DBP) or both SBP and DBP, which is, on repeated measurement, at or above the 95th percentile for age, sex and height. BP between the 90th and 95th percentile in childhood was designated "prehypertensive".

It further recommended that

1. “BP should be measured in the right arm with an appropriate cuff size(Flynn et al., 2017).
2. Appropriate steps should be taken to ensure the child is comfortable and still for at least 5 minutes prior to measurement.
3. Home BP measurements may help monitor hypertensives but not diagnose new cases.
4. Although measurement with an automatic BP cuff (oscillation device) may be used to screen for elevated BP in the primary care setting, it should be repeated with auscultation if the BP reading is elevated.
5. If an auscultated BP measurement is elevated despite correct measurement technique, it should be repeated two more times at the same visit and the average of the last two auscultatory readings used as a final BP. The degree of elevation should be assessed based on this average with reference to normative BP values for age, sex and height and the revised hypertensive categories as outlined in Figure 2A (Flynn et al., 2017).
6. A diagnosis of paediatric hypertension can be made if the BP remains at or above stage 1 hypertension on three separate clinic visits (Flynn et al., 2017). The timeline for these follow-up BP assessments is as outlined in figure 2A.
7. Compared to adults, their report gave no strict BP cut-off for a hypertensive crisis in children. However, it stated that severe hypertension warranting assessment in the emergency department depends on clinical symptoms, including irritability, headache, oedema, visual changes, or severe abdominal pain(Chandar & Zilleruelo, 2012; N. H. Patel et al., 2012; Stein & Ferguson, 2016).
8. Due to its superiority over isolated BP measurement in diagnosing hypertension and its ability to identify some secondary causes of hypertension, ambulatory blood pressure monitoring (ABPM) was recommended for screening or in confirming the diagnosis of hypertension if
   1. BP remains in the elevated BP category for ≥1 year
   2. BP remains in stage 1 hypertension category over three successive visits.
   3. The patient has high-risk conditions for hypertension (i.e., CKD, diabetes, obesity, or prematurity)
   4. Whitecoat hypertension is suspected”.


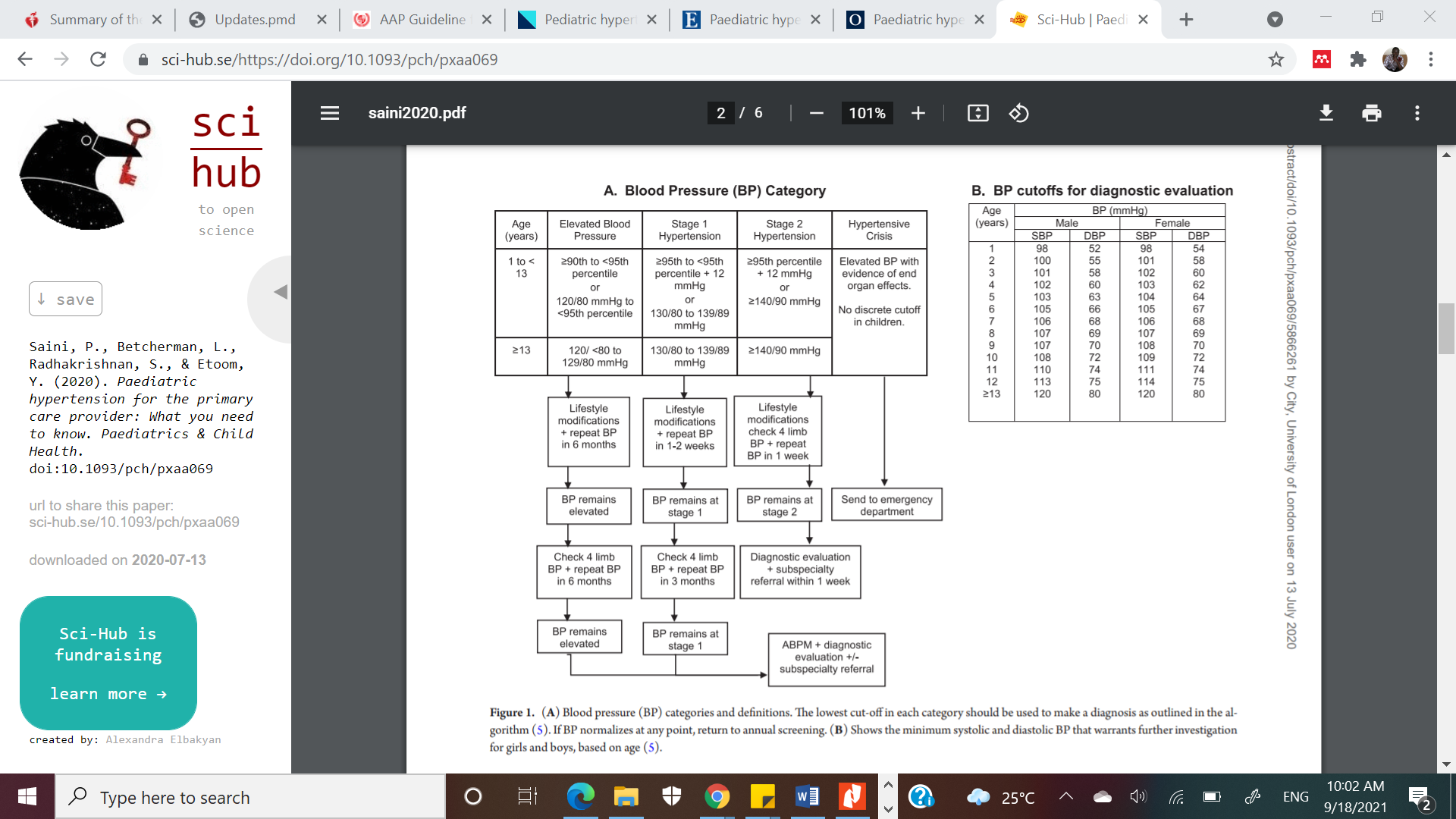


**Figure 2** (A) Blood pressure (BP) categories definitions and management algorithm (B) Minimum systolic and diastolic BP that warrants further investigation for girls and boys based on age(Flynn et al., 2017)

### **2.6.4 Investigating the aetiology of hypertension**

Because children and adolescents have a higher prevalence of secondary hypertension than the adult population (Falkner & Daniels, 2004), the guideline recommends a thorough screen for causative factors once a diagnosis of hypertension is made. This assessment should include a thorough perinatal, nutritional, physical activity, psychosocial, medical and family history. It should be followed by a complete physical examination to help provide clues to potential secondary causes of hypertension and to assess possible end-organ damage. It should include assessing the BMI, checking BP in multiple limbs to diagnose coarctation of the aorta, and other assessments based on the clinical history. To detect underlying secondary causes, laboratory evaluation of newly diagnosed children is recommended. Assessments for renal, endocrine and other conditions suggested by history and physical examination is also recommended. Clinicians are encouraged to screen for conditions that are likely to increase stress, including depression, anxiety, trauma, or bullying(Flynn et al., 2017). Additional recommended assessments include those for target organ damage. These include assessments for

1. “Central arterial stiffness using pulse wave velocity, cardio-ankle vascular index, carotid intima-media thickness and flow-mediated dilatation.
2. left ventricular hypertrophy using electrocardiogram or echocardiography
3. Renal and renovascular disease using renal ultrasound or CT scan, microalbuminuria and other biochemical tests, including serum uric acid.” (Flynn et al., 2017)

Beginning with the history, topics that should be addressed include the child's perinatal course, diet, exercise, obesity, medications, and family history. Social history warrants particular attention as family income, parental education, poor sleep, and a lack of physical activity are independent risk factors for increased BP among children and adolescents (Y. Shi et al., 2012).

A comprehensive physical examination should then be performed, covering each system involved in any differential diagnosis considered. Electrolytes, creatinine, lipid profile, blood urea nitrogen, and urinalysis are to be done for all children and adolescents with hypertension(Flynn et al., 2017).

### **2.6.5 First-line management of hypertension**

To prevent long-term sequelae of paediatric and adolescent hypertension and reverse end-organ damage, the guideline recommends a systematic approach to hypertension management once a diagnosis is made(see figure 1 A) (Flynn et al., 2017). The goals for management include targets for systolic and diastolic BPs.

In all cases, the first step in management involves lifestyle modifications, such as age-appropriate exercise and diet modifications(Flynn et al., 2017). For those within the category of elevated BP, lifestyle modifications should be implemented for approximately six months, after which the BP should be reassessed. Recommended target for BMI during this period is to have it <85^th^ percentile (Nerenberg et al., 2018). The indications for initiating pharmacotherapy include failure of lifestyle modifications, symptomatic hypertension, evidence of target organ damage, comorbid diabetes or chronic kidney disease, or stage 2 hypertension at presentation(Nerenberg et al., 2018; Y. Shi et al., 2012).

Lifestyle modifications should then continue to be used alongside pharmacotherapy, and regular follow-up appointments are recommended to monitor the BP until it is within target.

### **2.6.7 Referral**

Referral to a subspecialist should be considered for hypertension that does not respond to 6 months of lifestyle modifications, monotherapy-resistant, secondary or stage 2 hypertension, and symptomatic forms of hypertension. In remote areas where there might not be access to a paediatric nephrologist, clinicians are advised to consider telephone or telehealth consultation with a specialist. If urgent or emergent, transfer to the nearest centre with paediatric subspecialty care may be warranted(Saini et al., 2021).

### **2.6.8 Guidelines for screening and management of hypertension among children and adolescents in Ghana**

Guidance on the screening and management of hypertension among children and adolescents was first given in Ghana's sixth Standard Treatment Guidelines published in 2010(Andy, 2010). The recommendations in the document are similar to those in the Fourth Report for screening, diagnosis, investigation and management. Based on recommendations from the WHO, the Consolidated Guidelines for HIV Care in Ghana (2019) call on service providers to screen all persons living with HIV for hypertension at every visit and diabetes at set intervals. This is in line with WHO's guidance to integrate diabetes and hypertension care into HIV services as part of Differentiated Service Delivery in HIV care(WHO, 2020a). There is, therefore, a healthy policy environment to support the screening and management of hypertension among adolescents living with HIV in the country although they weren’t singled out in the HIV care guidelines.

## **2.7 Adherence to clinical practice guidelines**

Despite the efforts put into their development and dissemination, available evidence suggests a generally poor uptake of recommendations in CPGs, with reported adherence ranging from 0% to 98.9%. Despite wide promulgation, clinical practice guidelines have had limited effect on changing physician behaviour (Cabana et al., 1999). Evidence indicates that all health professionals vary in the extent to which they follow CPGs and that this causes unsafe interventions and unsafe practice(Davies et al., 2008; Puffer & Rashidian, 2004; Y. Shi et al., 2012).

Two years after the release of a guideline on the management of hypertension, a survey among Physicians in New Zealand showed that only 40% had read the guideline(Arroll et al., 1995). Although 78% of the Physicians surveyed by Rosser indicated that they complied with the guidelines, further questioning revealed that only 5% of the respondents followed them(Rosser & Palmer, 1993).

### **2.7.1 Adherence to Guidelines for screening and management of hypertension among children and adolescents**

Despite the publication of the Fourth Report and other guidelines from Canada and Europe for diagnosis and management of hypertension among children and adolescents(De Jesus, 2011a; Hagan et al., 2007; Lurbe et al., 2019), a review by Shapiro revealed that between 2000 and 2009 in the United States, healthcare providers checked the BPs of adolescents at only a one-third of their out-patient clinic visits(Shapiro et al., 2012). Records review by Hansen and colleagues found only 26% of the 507 hypertensive adolescents at a facility to be appropriately diagnosed(Hansen et al., 2007).

### **2.7.2 Factors affecting HCW adherence to guidelines**

In their systematic review of theoretic concepts, practical experience and research evidence in the adoption of clinical practice guidelines, Davis and Taylor found out that the adoption of guidelines is influenced by the demographic characteristics of the health care professional, characteristics or culture of the practice setting, incentives( legal or financial), practice regulation concerning the guideline, patient factors and the qualities of the guidelines concerning its relative advantage, compatibility with existing beliefs and values, its complexity, "trialability," "observability" and the cost of adherence(Davis & Taylor-Vaisey, 1997).

Their finding was supported by Cabana's review on barriers to physician adherence to clinical practice guidelines, which identified the influence of critical determinants of knowledge and attitude and their translation into guideline adherence behaviour(Cabana et al., 1999).

According to them, knowledge as a barrier to CPG adherence results mainly from lack of awareness of the CPG’s content. This, was attributed to the volume of information in the document, time needed to stay informed, and accessibility to the document. Healthcare worker attitudes as a barrier to CPG adherence, according to the authors, was due to lack of outcome expectancy for the recommendations, lack of self-efficacy to adhere to the recommendations and lack of motivation to adhere due to previous practices, habits and routines. Attitude as a barrier was also said to result from lack of agreement with the guideline recommendations due to a perceived wrong interpretation of the evidence, the applicability of recommendations to patients, cost-benefit assessment for adherence, and lack of confidence in the guideline developer.

In addition, they reported patient preference, presence of contradictory guidelines and environmental factors such as lack of time, lack of resources to support adherence and organizational constraints as external barriers to CPG adherence behaviour (Cabana et al., 1999).

In a survey by Tammy and colleagues, 87% of children with elevated BPs were not recognized(Brady et al., 2010), and they found patient-, provider- and clinic-level factors predicting the under-recognition of the BPs. Patient-level predictors of under-recognition included systolic BP of <120 mmHg (odds ratio: 7.7 [95% confidence interval: 3.2-18.6]), diastolic BP of <80 mmHg (odds ratio: 2.4 [95% confidence interval: 1.1-5.0]), decreasing BMI z score, male gender, older age, lack of family history of cardiovascular disease, and negative medical history findings. Being seen by a nurse practitioner or a less-experienced provider were also a significant predictor. Variability in the BPs due to variations with age, sex and height were also blamed for the missed diagnosis(Rinke et al., 2019). A survey of 89 pediatricians found that more than half of them were not familiar with the most current published recommendations for diagnosing and treating pediatric hypertension, and this was the most common reason for not initiating pharmacotherapy for children with hypertension followed by concerns for adverse medication effects(Boneparth & Flynn, 2009).

## **2.8 Theoretical determinants of healthcare worker behaviour towards clinical practice guidelines**

Several theoretical frameworks have been used to explore the healthcare worker behaviour towards clinical practice guidelines. These frameworks help evaluate a set of beliefs and understand cognitive and behavioural responses, thus helping to predict healthcare worker behaviour (Godin et al., 2008). According to Yami(Yami, 2015), the most frequently used psychological theories to examine behavioural intentions across a wide range of health issues include the Health Belief Model(Rosenstock, 1974), Social Cognitive Theory(Bandura, 1986), the Theory of Reasoned Action(Ajzen & Fishbein, 1985) and the Theory of Planned Behaviour(TPB)(Ajzen, 1991). However, the most commonly used is the TPB(Yami, 2015).

### **2.8.1 The Theory of Planned Behavior (TPB)**

The TPB is an extension of the TRA proposed by Fishbein and Ajzen(Thompson, 2014). Through the TRA, they sought to understand behaviour by looking at the relationship between attitudes, subjective norms and behavioural intentions. Attitudes and subjective norms were posited as determinants of intention(Ajzen & Driver, 1991; Icek Ajzen, 1980). It was then presumed that intention directly influences behaviour. However, many researchers suggested TRA was insufficient when individuals believed they had little control over their decisions(Ajzen, 1991). Therefore, the TPB emerged, having attitude, subjective norms and Perceived Behavioral Control (PBC) as constructs and determinants of intentions to undertake a behaviour (Ajzen, 1991). Therefore, the concept of non-volitional factors (i.e. perceived behavioural control) was added to the TRA to form the TPB, making it explore the understanding of human behaviours under both volitional and non-volitional control(Ajzen, 1991).

#### **Attitude**

Attitude refers to the individual's overall evaluation of their behaviour, which might be positive or negative(Ajzen & Fishbein, 1985). Attitudes are established from a combination of beliefs about behaviours and an evaluation of their outcomes(Ajzen, 1991). Therefore, the attitude construct assesses beliefs toward behavioural performance outcomes that can be weighted by the individual's positive or negative evaluations(Ajzen, 1991).

#### **Subjective norms**

Subjective norm refers to an individual's perception of others' beliefs (Icek Ajzen, 1980). Subjective norms depend on normative beliefs; people's desire and willingness to receive commendation and support from others influence their motivation to adhere(Ajzen & Fishbein, 1985). Normative beliefs interact with multiple factors to encourage compliance. Specific individuals, therefore, need to be emulated to determine subjective norms. Thus, the more favourably an individual evaluates behaviour, and the stronger the perception of support from others, the stronger they will intend to do the behaviour and the more likely the person is actually to engage in that behaviour (Ajzen, 1991).

#### **Perceived Behavioural Control**

The perceived behavioural control construct assesses an individual's actual possession of the opportunities and resources required to perform the desired behaviour (Ajzen & Fishbein, 1985; Ajzen, 1991). It provides significant and consistent evidence distinguishing between control beliefs and perceived power(Icek Ajzen, 1980). Control beliefs focus on possibilities that inhibit or simplify the behaviour, and perceived power focuses on psychological constraints that inhibit or simplify the performance of the behaviour (Ajzen, 1991). According to Bandura, PBC comprises an internal factor, i.e. the self-efficacy concept, representing the perception of individual capability, and an external factor representing the perception of control over environmental barriers. Apart from its indirect effect on behaviour by influencing intentions, the theory postulates that perceived behavioural control can directly affect behaviour. (Bandura, 1986)


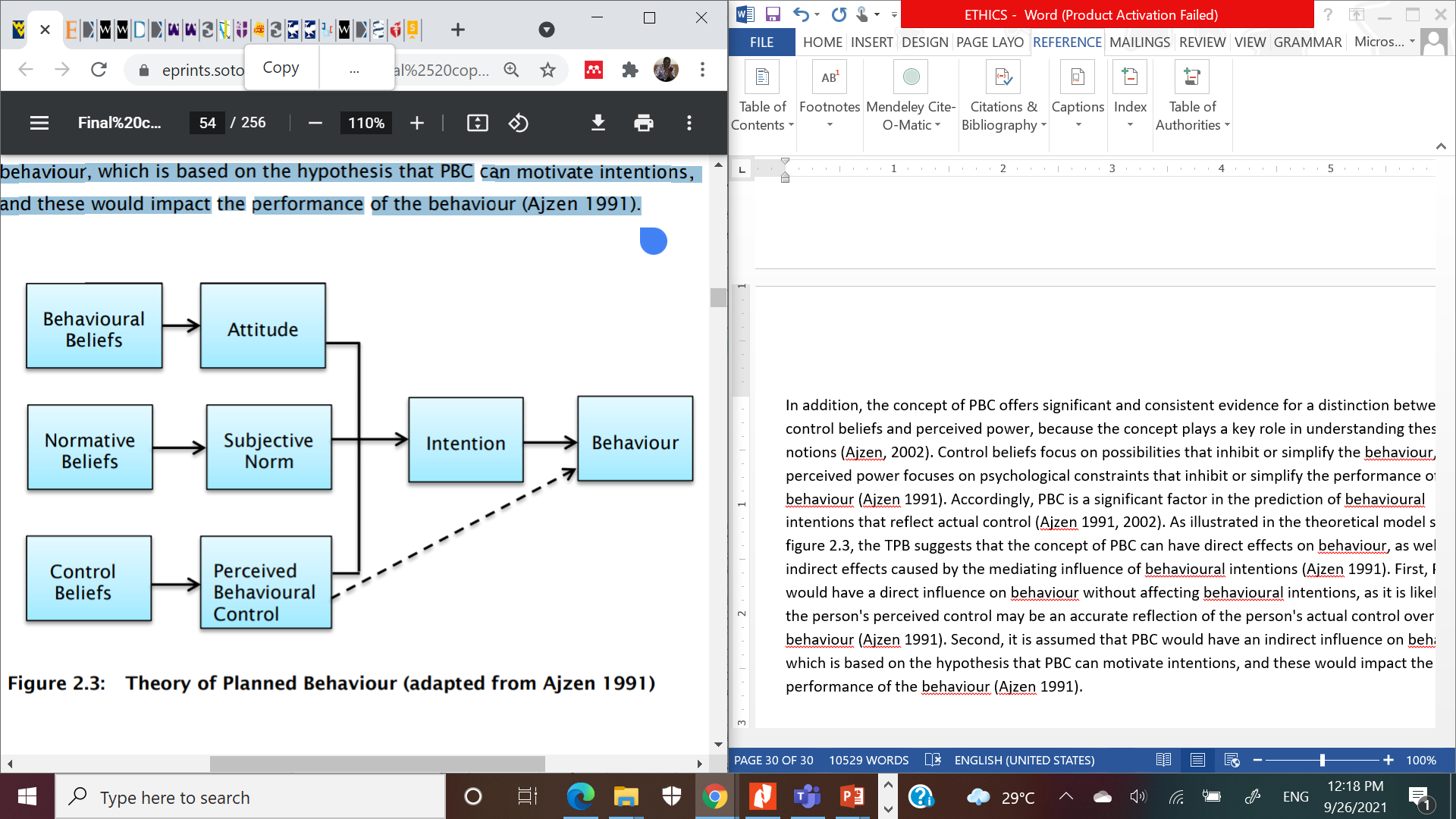


**Figure 3 Theory of Planned Behaviour(Ajzen, 1991)**

In general, the TPB variables (attitude towards the behaviour, subjective norms, and PBC) determine the influence of the behavioural intention that leads individuals to perform the actual behaviour or not to perform that behaviour (Figure 3). According to the theory, HCW adherence to guidelines will depend on how favourable they assess the guidelines, how they feel socially pressured to adhere, and whether they are equipped to adhere to them.

### **2.8.2 Previous guidelines adherence research among healthcare workers using TPB**

In a cross-sectional survey by Kortteisto and colleagues among HCW in a Finnish organization, the TPB explained 36% of the variations in their intention to adhere to CPG in their various speciality areas(Kortteisto et al., 2010). Among Nurses in Quebec, it explained 50.3% of their intentions to use needle filters(Gagnon et al., 2015). Nelson also used the theory to examine the behaviour of Nurses and Physician Assistants towards BP monitoring and found that only the intention construct of the model predicted their actual practice(Nelson et al., 2014).

In another study in Taiwan, Ko and colleagues used the TPB to explore nurses' behavioural intentions to comply with the protocol of managing occupational exposure to blood and body fluids(Ko et al., 2011). Their findings showed that the model accounted for 54% of the variance in nurses' behavioural intentions and that the PBC (β =0.58), subjective norm (β =0.15) and attitude (β =0.12) were significant constructs and had direct influences on nurses' behavioural intentions to comply with post-exposure management.

In an exploratory mixed methods study by Puffer and Rashidian to examine the utility of the TPB model in predicting and explaining nurses' behavioural intentions to comply with clinical guidelines in providing smoking cessation advice, the model explained 40% of the variance in their behavioural intentions. The authors found PBC(r=0.55) and attitude( r=0.42) to have the strongest relationship with behavioral intentions(Puffer & Rashidian, 2004).

These and other findings from meta-analytic reviews(Bobo Kovač & Rise, 2011; Webb & Sheeran, 2006) make the TPB model an appropriate and reliable theoretical framework for predicting behavioural intentions and justify the selection of the TPB for this current study, which will examine healthcare workers' behavioural intentions towards compliance with the guidelines for screening and management of hypertension among adolescents.

## **2.9 Interventions to improve healthcare worker adherence to clinical practice guidelines**

A systematic review by Davis and Taylor identified four main strategies used to facilitate the implementation of guidelines. They included traditional Continuous Professional Development Programmes (CPD), community-based interventions, practice-based interventions and the use of multiple-intervention strategies(Davis & Taylor-Vaisey, 1997).

### **2.9.1 Continuous Professional Development Programmes**

In their review, the CPD approaches identified included mailed materials, formal conferences and workshops. While Oakeshott, Kerry and Williams used mailed educational materials to improve general practitioners' ordering of radiographs (Oakeshott et al., 1994), formal conferences, courses, symposia, workshops and small-group discussions had inconsistent results (Davis & Taylor-Vaisey, 1997). Whiles Karuza and colleagues used small group discussions to improve pre-operative hand washing among health workers, Brown and his associates found little or no improvement in cholesterol management after a 3-hour seminar, enhanced by follow-up meetings and printed materials(Jeffery et al., 2015; Karuza et al., 1995).

### **2.9.2 Community-based interventions**

Nardella used academic detailing to significantly reduce test ordering among Nurse Practitioners(Nardella et al., 1994). Lomas and colleagues also used opinion leaders to improve adherence to guidelines for vaginal birth after a previous caesarian section(Lomas et al., 1989).

### **2.9.3 Practice-based interventions**

Patient-based interventions, audit and feedback, and reminders constituted the review's practice-based interventions. Patient-based educational interventions involving educational materials for diabetes and smoking cessation helped improve healthcare worker adherence through increased patient demand(Mazzuca et al., 1990; Wilson et al., 1988). Audit and feedback interventions have been shown to have mixed effects on healthcare worker behaviour. Robinson suggested that it is more effective when given concurrently than later and retrospectively(Robinson, 1994). Johnson and Martin also recommended using provider-specific feedback to maximize healthcare worker adherence behaviour (Johnson & Martin, 1996). On the use of reminders, Dartnell and colleagues used posters and pocket-sized laminated cards to successfully augment the dissemination of anticoagulation guidelines on hospital wards(Dartnell et al., 1995).

#### **Multiple-intervention strategies**

Clinical practice guideline dissemination strategies involving two or more interventions have impacted healthcare worker behaviour and clinical outcomes than single interventions(D. A. Davis et al., 1995; Oxman et al., 1995). Benninger, King, and Nichols used mailed materials, follow-up phone calls and presentations to decrease inappropriate otolaryngology referrals.

Therefore, a multi-component intervention based on the theory of planned behaviour will be employed for this study.

## **2.10 Gaps in the literature**

Ghana introduced the guidance for screening and management of hypertension among children and adolescents in the sixth Standard Treatment Guidelines (STG) (2010). This was a significant intervention because adolescents in Ghana are at increased risk of elevated blood pressure. In addition to obesity and other traditional risk factors driving adolescent hypertension in the country, in utero exposure to malaria has increased the odds of hypertension during adolescence(Ayoola et al., 2014; Etyang et al., 2019). A cohort study in Ghana by Bedu Addo et al. showed, after controlling for other factors, that the children of mothers who had malaria during their pregnancy had 2.23mm (1.50, 5.97) and 2.10mm (0.95, 5.16) higher systolic and diastolic blood pressures respectively in adolescence, compared to the children of uninfected mothers(p< 0.05)(Bedu-Addo et al., 2017). With Ghana being an endemic malaria country, surveillance of the blood pressures of adolescents is paramount.

In addition to this, Ghana has about 23,000 adolescents living with HIV as of December 2020(UNAIDS, 2021a), with over 80% of them perinatally infected, thus having more prolonged exposure to the virus and the metabolic effects of antiretroviral medication. The country's adoption of the latest WHO guidelines for antiretroviral therapy(WHO, 2021a) allows introducing dolutegravir- and lopinavir-based regimens very early in life, further increasing their risk of being hypertensive in adulthood. All these contributed to the call by WHO for integration of hypertension management into HIV care as part of differentiated HIV service delivery(BeLue et al., 2009; P. Patel et al., 2018; UNAIDS, 2008; WHO, 2020a).

Despite the high number of at-risk adolescents and the call by the WHO, the healthcare workers' level adherence to the STG on screening adolescents for hypertension is unknown(Andy, 2010). To contribute to the global target of reducing hypertension by 25%, this information is critical to guide efforts and policies at improving case detection, primary prevention and case management. These will help reduce the persistence of elevated adolescent blood pressures into adulthood, reduce the country's hypertension and CVD burden, and decrease the potential negative impact it would have on the country's health system and economy.

## **2.11 Conceptual Framework**

The PRECEDE-PROCEED planning Model will guide the conduct of the study. The PRECEDE (Predisposing, Reinforcing, and Enabling Constructs in Educational Diagnosis and Evaluation) phase will help with the planning whilst the PROCEED (Policy, Regulatory, and Organizational Constructs in Educational and Environmental Development) phase guides the implementation(RHIhub, 2018). The theory of planned behaviour has been embedded in the PRECEDE-PROCEED model, as found in figure 4, to form the conceptual framework for the study.

**Clinical Practice Guideline dissemination and regulation**

**Attitude towards adolescent BP screening**

**Subjective norms around Adolescent BP Screening**

**Perceived** **behavioral** **control towards adolescent BP Screening**

**Health care worker intention** **to** **adhere to guideline**

**Health care worker adherence** **to** **guidelines for Adolescent hypertension**

**Prevention, diagnosis and management of adolescent hypertension**

**Reduced** **incidence** of **adolescent** **hypertension**

**Reduced** **arterial** **stiffness**

The intervention in the conceptual framework is the dissemination of clinical practice guidelines and equipping service providers to adhere. This is expected to improve the knowledge and attitude of healthcare workers towards the screening of adolescents for hypertension, change their perceived subjective norms concerning the checking of BPs among adolescents and improve their perceived behavioural control and self-efficacy to screen adolescents for hypertension and manage them appropriately. Together, these are expected to increase their willingness or intention to adhere to the guidelines, translating into the adherence behavior. Adherence to the guidelines by HCW will improve the prevention, diagnosis and management of adolescent hypertension. This will decrease the ARV-induced arterial stiffness of adolescents living with HIV and decrease their likelihood of being hypertensive. It will also decrease the blood pressures of adolescents, preventing them from being hypertensive and developing arterial stiffness.

# **CHAPTER 3: METHODOLOGY**

## **3.1 Introduction**

This section will describe the study design and operationalization, sampling, data collection and analysis procedures. In addition, the section discusses procedures to protect human subjects. To achieve the objectives of the study, non-experimental and experimental approaches will be employed. The non-experimental component of the study will involve the conduct of a cross-sectional survey to determine factors influencing healthcare workers’ intention to adhere to the guidelines for screening and management of hypertension among children and adolescents. The experimental component of the study will assess the effect of a theory of planned behaviour-based intervention on healthcare worker adherence to the guidelines for screening and management of hypertension among adolescents living with HIV, using a cluster-randomized study.

The non-experimental and experimental components are described below.

## **3.2 Non-experimental component**

### **3.2.1 Behaviour of Interest**

The survey will assess healthcare workers’ intention to adhere to the guidelines for screening and managing hypertension among children and adolescents. The proxy behaviour to be assessed during the survey is their practice of screening and interpreting the blood pressures of adolescents (10-17 years) living with HIV during every clinical visit.

### **3.2.2 Study design**

The study will employ a cross-sectional design to describe healthcare workers’ intentions to screen adolescents for hypertension. It will also describe the relationships between the constructs within the theory of planned behaviour (TPB) model. The study will be undertaken in two phases. The first phase will involve the development of a self-administered TPB-based questionnaire to be used for a cross-sectional survey in the second phase. The questionnaire development process will be guided by the “manual for constructing questionnaires based on the theory of planned behaviour” (Francis et al., 2004) and will involve an elicitation study, questionnaire formatting and validity assessment for the questionnaire.

Staff from ART facilities having 20 to 40 adolescents on treatment will be engaged to support the questionnaire development process in the first phase while those from facilities with at least 40 adolescents living with HIV on treatment will be targets for the second phase of the study.

The phases of the non-experimental study are now described.

#### **3.2.3 Elicitation study**

The first step is to determine the predictors of adolescent blood pressure screening by eliciting healthcare workers’ behavioural, normative and control beliefs about checking and interpreting the blood pressures of adolescents.

1. **Study Settings**

The elicitation study will be conducted among clinical health workers in the following ART sites that will not take part in the second phase of the study:

- Dansoman Polyclinic
- Amanfro Health Centre
- Pentecost Hospital
- Botianor Health Centre
- Sukura Community Clinic

1. **Sampling**

In each facility, participants will be randomly selected from eligible healthcare workers using labelled papers (Yes/No) in an opaque envelope. Those who select yes will be eligible for inclusion. Prior to the balloting, prospective participants’ selection will be based on the following inclusion and exclusion criteria**:**

**Inclusion criteria**

- Registered clinical staff with valid licenses from the relevant regulatory bodies in Ghana. They may include Doctors, Physician assistants, Pharmacists, Nurses or Midwives
- Registered staff providing antiretroviral services to clients or working at the outpatient department of the health facility
- Aged 18 years or over
- Must have practical post-qualification experience of at least one year.

**Exclusion criteria**

- Does not consent to part take in the study

1. **Sample size**

Francis and colleagues recommended that at least 25 participants be included in any elicitation phase of research using an open-ended questionnaire(Francis et al., 2004). With an anticipated 50% response rate as observed in a similar study (2), at least 50 participants will be enrolled in this elicitation study.

1. **Elicitation Of Behavioral, Normative And Control Beliefs**

As recommended in the manual for constructing questionnaires based on the theory of planned behavior(Francis et al., 2004) and adapted for this study, the questions to be used to elicit the health workers’ behavioral, normative, and control beliefs can be found in the appendix.

1. **Analysis**

Two individuals, including the PI, will analyze the content of the responses by developing themes, labelling them and listing them in order of frequency. The most frequently reported beliefs, described as the modal set, will be used to develop the instrument to measure the health workers’ behavioural, normative, and control beliefs. As recommended, at least 75% of all beliefs listed will be included in the tool to cover the belief’ population” adequately.

#### **3.2.4 Formatting the Questionnaire**

A draft questionnaire will be developed based on the manual for constructing questionnaires based on the theory of planned behavior(Francis et al., 2004) using responses from the elicitation study. As Francis(1) recommended, the final questionnaire will contain a minimum of 40 carefully worded items across the seven theory of planned behavior domains and eight demographic items.

#### **3.2.5 Assessment of Validity and Reliability**

Five respondents will be asked to complete the draft questionnaire to help provide feedback on the ambiguity or difficulty of the questions, the length, wording or formatting of the questionnaire and inconsistency of responses. In addition, comments from experts, consultants and researchers in clinical practice and behavioural sciences on the draft questionnaire will be used to determine the construct validity of the tool. The reliability of the questionnaire will be ascertained by determining its internal consistency (Cronbach’s coefficient alpha) using STATA version 16.

### **3.2.6 TPB-Based Survey among Health Workers**

#### **a. Study Setting**

The survey will be conducted among health workers in the top 20 ART sites in the Greater Accra Region with the highest Adolescent HIV client load. According to the National AIDS Control Programme, these were the facilities as of December 2021:

- Tema General Hospital
- International Health Care Centre
- Ussher Polyclinic
- Ga South Municipal Hospital
- Ridge Regional Hospital
- Princess Marie Louis Hospital
- Ashaiman Polyclinic
- LEKMA Hospital
- Kaneshie Polyclinic
- Ga West Municipal Hospital
- Mamprobi Polyclinic
- 37 Military Hospital
- Achimota Hospital
- Madina Polyclinic(Kekele)
- Maamobi General Hospital
- Tema Polyclinic
- Adabraka Polyclinic
- Legon Hospital
- Pantang Hospital
- Amanfro Health Centre

#### **b. Sampling**

In each facility, participants will be randomly selected from eligible healthcare workers using labelled papers( Yes/No) in an opaque envelope. Those who select yes will be eligible for inclusion. Prior to the balloting, prospective participants selection will be based on the following inclusion and exclusion criteria**:**

**Inclusion criteria**

- Registered clinical staff with valid licenses from the relevant regulatory bodies in Ghana. They may include Doctors, Physician assistants, Pharmacists, Nurses or Midwives
- Registered staff providing antiretroviral services to clients or working at the outpatient department of the health facility
- Aged 18 years or over
- Must have practical post-qualification experience of at least one year.

**Exclusion criteria**

- Does not consent to part take in the study

#### **c. Sample Size**

This survey includes seven TPB variables. To detect a medium effect size of 0.15 with a power of 80% and alpha of 0.05 as Yami(2) did, a power analysis using STATA version 16 gave a minimum sample size of 89 respondents. With an anticipated 50% response rate as found in a previous study(Yami, 2015), at least 180 respondents will be invited to participate in the survey.

#### **d. Recruitment**

The administrators of prospective facilities will be engaged to seek their consent for facility participation. The study will be introduced to healthcare workers in the consenting facilities by the PI during their weekly clinical meetings. An anonymous questionnaire pack with an invitation letter and consent form will be left with the heads of relevant clinical units in the health facilities so they support and encourage the participation of their team members. A drop box will be left at the administration of each facility for completed questionnaires to be dropped in. Weekly reminders will be provided to all prospective respondents to ensure they respond within the survey period. For participants who would like to respond electronically, the link to a google form containing the questionnaire will be shared with them for completion.

#### **e. Data collection**

Data collection will be done using the close-ended self-completion questionnaire developed during phase one of the study. Printed copies and an electronic form of the questionnaire will be available. Data collection will be done within eight weeks.

#### **f. Data analysis**

Data will be exported into STATA version 16 for analysis. Descriptive statistics (frequencies and percentages) will be used to present demographic variables and participants’ characteristics. The internal consistencies of each TPB construct in the questionnaire will be verified by calculating Cronbach’s alpha. To determine the strength of relationships among all variables, correlation coefficients will be employed. Multiple regression analysis will be used to determine the extent to which the attitudes, subjective norms and PBC variables (independent variables) can predict healthcare workers’ intentions to comply with the guidelines for screening and management of hypertension among adolescents living with HIV.

## **3.3 Experimental Component**

This study component will assess the effect of a multi-component theory of planned behaviour-based intervention on healthcare worker adherence to the guidelines for screening and management of hypertension among children and adolescents.

### **3.3.1 Study Design**

This will be a multi-facility, two-arm cluster-randomized study and will follow the CONSORT guidelines for the conduct of cluster randomized studies(CONSORT, 2010). There will be random allocation by clusters (health facilities) to the control and intervention arms of the study.

### **3.3.2 Study Settings**

The study will be conducted in antiretroviral therapy facilities in the Greater Accra Region. Facilities with a minimum of 40 adolescent clients on treatment as of December 2021 will be eligible for inclusion. However, facilities that do not consent to participate and those participating in other studies involving the ART staff or the adolescents living with HIV will not be included.

### **3.3.3 Participants**

The primary beneficiaries of the study are the health workers in the prospective facilities. The secondary beneficiaries are the adolescents living with HIV who assess antiretroviral care in these facilities. The beneficiary population to be included in the study are described below:

1. **Healthcare workers**

Registered clinical staff in consenting health facilities with valid licenses from relevant regulatory bodies in Ghana will be eligible for inclusion in the study. They may include prescribers, Pharmacy staff, Nurses and midwives. They will, however, be excluded if

- Less than 18 years of age
- They do not provide antiretroviral services to clients or work at the outpatient clinic
- Their practical post qualification experience in providing client care is less than one year.
- They are not available throughout the study period
- They do not agree to participate in the study by signing a consent form.

1. **Adolescents living with HIV**

Adolescents living with HIV who are clients in the study facilities will all be eligible for inclusion in the study. They will, however, be excluded if

1. They have any stage 3 or 4 HIV defining conditions
2. They started antiretroviral medications less than six months prior to the study enrolment
3. They have been diagnosed with mental illness or have a cognitive deficit
4. They are participating in another experimental study
5. There is a lack of parental or participant consent or participant assent to participate in the study.

### **3.3.4 Recruitment**

Recruitment into the study will be done at three levels: health facilities, health workers and adolescents living with HIV.

1. **Health facility recruitment**

The PI will invite potential participating clusters (i.e. health facilities) through their respective administrators to participate. In each consenting facility, the PI will identify a primary contact person for the research team.

1. **Clinical staff recruitment**

Once facilities are recruited, the PI will contact the focal persons to obtain details of potential clinical staff who may be interested in participation based on the inclusion and exclusion criteria. The potential participants will be engaged to discuss the study protocol. Those who express interest will be provided with a study invitation pack, including a letter of invitation, information sheet and consent form.

1. **Patient recruitment**

Using a random sequence provided by the study team, a random sample of 20 adolescents living with HIV per facility will be selected for baseline and end-line assessment. The consent of their caregivers will be sought, followed by the adolescents’ consent or assent as may be age-appropriate.

### **3.3.5 Sample size**

This study is designed to detect a doubling (100%) of the proportion of adolescent visits that had BP screening done (conservatively calculated in STATA version 16 as an increase from 17% to 34%) between the intervention and control groups. It is based on the assumption of a 2-sided test with 80% power at a 5% significant level, cluster sizes of 20 adolescents and an intra-cluster correlation coefficient (ICC) of 0.05. Accordingly, two groups of ten (10) health facilities will be needed at the minimum. As this sample size estimation does not account for any potential loss to follow-up, we will recruit additional two facilities per arm (24 in total). Thus, a total of about 96 health workers (4 per facility) and about 480 adolescents living with HIV (20 per facility) will be enrolled in this study.

### **3.3.6 Randomization**

**a. Sequence generation:** The unit of randomization will be the health facility. Randomization will be performed by a statistician independent of the study team, using computer-generated random permuted blocks and will be stratified by type of health facility (regional, district/ municipal, Polyclinic, Health Centre). Twelve facilities will be in the intervention arm and the rest in the control arm. The study statistician will provide each facility with a computer-generated list of random numbers to select a sample of 20 adolescent clients to participate in the baseline and end-line survey.

**b. Allocation concealment**: Each facility will eventually become aware of its allocation, but allocation will be concealed until the baseline assessment is completed. Adolescents will not be allocated to alternative interventions within a facility, so allocation concealment will not be an issue.

**c. Implementation of clusters:** The PI will provide the list of prospective facilities by type to the study statistician, anonymized by an ID**.** The study statistician will generate the random sequence and allocate practices to two separate dummy-coded groups without knowing which group will receive the intervention; the study statistician will remain blind to allocation until the collected follow-up data. Random allocation of clusters to intervention or control will occur consecutively as practices are recruited until the target sample size (24) is reached.

**d. Blinding:** Given the nature of the intervention, healthcare workers will inevitably be aware of their allocation and thus, blinding participants will not be possible. Each facility will also become aware of allocation. However, facilities will not be informed of allocation until after both facilities and individual health workers are identified and recruited (i.e., not until after baseline assessment). Adolescents within each facility will remain blind to allocation. The outcome assessors will be kept blind to the allocation. The facility contact that will use the random sequence of numbers to identify the sample of 20 patients within each facility will possibly not be blind to allocation. Blinding the entire research team is also impossible as intervention facilities will be contacted to arrange the sessions. However, the study statistician conducting the outcome analysis will remain blinded to allocation until after the outcome data have been collected at follow-up.

### **3.3.7 Intervention**

The 12 intervention facilities will receive a multi-component intervention package based on the theory of planned behaviour as captured in ***table 3.1***. They will be oriented on the risk of hypertension among adolescents living with HIV, be provided monthly feedback on their performance for the initial three months and receive the support of an opinion leader in their facility. They will also be provided with clinical decision support and sphygmomanometers with paediatric cuffs. The Fidelity of intervention delivery will be assessed using the process measures captured in ***table 3.1*** across the TPB domains.

### **3.3.8 Comparator**

All 24 facilities will be given an orientation on the guidelines for screening and managing hypertension among children and adolescents. This will be done by trained facilitators using PowerPoint presentations, group discussions and case scenarios.

**Table 1** **Application of Theory of Planned behavior to intervention design and measurement**

| **TPB Constructs** | **Intervention package** | **Process Measures** |
| --- | --- | --- |
| Attitude | 1. Orientation on hypertension risk among adolescents living with HIV | Pre and post-test scores |
| Subjective norms | 1. Audit and feedback 2. Opinion leaders 3. Patient education 4. Reminders(posters) | 1. The proportion of planned feedback sessions undertaken 2. The proportion of planned engagement sessions with opinion leaders held 3. The proportion of planned patient education sessions held 4. The proportion of expected posters pasted |
| Perceived behavioral control | 1. Provision of a paediatric sphygmomanometer 2. Orientation on BP measurement technique 3. Provision of clinical decision support | 1. The proportion of intervention facilities with paediatric sphygmomanometer 2. a.Proportion of target staff oriented on BP measurement technique   b. change in accuracy of BP measurements post-orientation   1. The proportion of intervention facilities with clinical decision support pack |

### **3.3.9 Outcomes**

#### **a. Primary outcomes**

The primary outcome will be the effect of the intervention on healthcare workers’ adherence to the guideline. It will assess adherence to recommendations on the

1. frequency of screening
2. Detection of elevated or high BP,
3. Investigation of high BP and
4. Management of elevated or high BP.

These will be assessed using a data extraction tool based on the HIV client care booklet. The tool will extract the data from the client care records of adolescents who received care within the period of interest. At baseline, it will assess the records of 20 randomly selected adolescents who received care at the facility six months prior to the assessment and extract the data from their most recent visit. At follow up, data will be extracted from the most recent visit of 20 randomly selected adolescents who received care at the facility after the intervention commenced.

#### **b.Secondary outcomes**

The secondary outcomes of the study will assess the effect of the intervention on the adolescents living with HIV at the intervention facility. A cross-sectional survey and physiological assessments will be conducted among 20 randomly selected adolescents per facility at baseline and six months after the intervention in both arms of the study.

1. **Cross-sectional Survey among Adolescents Living with HIV**

This survey will validate the data extracted from the records and confirm that the adolescents received the interventions. Data will be collected by face-to-face interviews using a structured questionnaire based on an adaption of the WHO STEPS Questionnaire for Chronic Disease Risk Factor Surveillance(WHO, 2012). The questionnaire will collect data on; socio-demographic characteristics, history of tobacco use and alcohol consumption, physical activity, diet and anthropometric measurements. In addition, data will be collected to assess the

- Number of clients who reported having their BPs checked during the last clinical visit
- Number of clients who reported being educated on hypertension and its risk reduction during the last clinical visit and
- The number of clients who reported adhering to any preventive measures (diet, physical activity) in the week prior to the interview.

Height and weight measurements of respondents will be taken with calibrated stadiometers and a weighing scale, respectively. All measurements will be taken using the US Centers for Disease Control and Prevention (CDC) standard procedure(CDC, 2014).

1. **Physiological Assessments**

These assessments will identify adolescents with normal blood pressure, masked hypertension, white coat hypertension, elevated BP and high BP as defined in the guideline for screening and managing hypertension among children and adolescents(Flynn et al., 2017). The assessment will also help identify those at risk of being hypertensive (having arterial stiffness). These will be achieved by measuring their office blood pressure, ambulatory blood pressure and cardio-ankle vascular index (arterial stiffness) as described below.

- **Office Blood pressure**

Participants’ blood pressure will be assessed as recommended in the guideline for screening and managing hypertension among children and adolescents(Flynn et al., 2017) and described in section **2.6.3.**

- **Ambulatory blood pressure measurement(ABPM)**

This will be performed using the CONTEC ABPM50 24H Ambulatory Blood Pressure Monitor. It will be done over 24 h, with BP readings every 30 min. A minimum of 70% accurate readings will be considered sufficient. Wake and sleep periods will be based on sleep and wake times recorded in the participant’s journal. Height-specific ABPM percentiles presented in the guidelines(Flynn et al., 2017) and associated references will be used to define ABPM abnormal BP threshold values and percentiles and to define ABPM-specific BP abnormalities(Fredric et al., 2021).

- **Cardio-Ankle Vascular Index**

This will be assessed using the VaSera 1500N vascular screening system (Fukuda Denshi Co.). As recommended, examinations will be conducted under standard conditions (room temperature 22 °C, minimization of stimuli) between 8.00 and 10.00 a.m. Participants will be instructed to avoid physical exercise and consumption of substances that could affect cardiovascular function (e.g., caffeine, alcohol) 24 hours prior to the examination. Before the examination, participants will rest in a supine position for 15 minutes to avoid the potential effects of stress. At the time of examination, subjects will remain supine with both arms on the bed, parallel to the body, and limb cushions placed below the elbows and heels to prevent contact of the limb cuffs with the bed. Electrocardiographic electrodes will be placed on both wrists, oscillometric blood pressure (BP) cuffs wrapped around the arms and ankles, and a microphone for phonocardiography placed on the sternum at the level of the second intercostal space. The cardio-ankle vascular index will be measured to determine the presence of arterial stiffness based on age- and sex-defined cut-offs(Rico Martín et al., 2020).

## **3.4 Data Management**

### **3.4.1 Data Quality Control**

Qualified research assistants will be trained in collecting data by conducting pre-tests of the questionnaires and other data collection tools. The PI will cross-check all data entry forms to ensure errors are corrected. All data will be entered in duplicate to reduce data entry errors. Hard copies of data will be stored under lock and key. Soft copies of data will be stored in a One drive account accessible to the principal investigator only.

### **3.4.2 Data Analysis**

Data will be analyzed using STATA 16. Summaries of variables will be obtained using frequencies, means and standard deviations. Histograms, bar graphs and box and whisker plots will be used to check for skewness and identify outliers. Proportions and frequencies will be used to summarise all binary or categorical variables. Means and standard deviations will be used to summarise continuous variables and proportions and frequencies for categorical variables. Chi-square tests will be used to determine associations between categorical variables. Independent sample t-tests or one-way ANOVA will be used to test for differences in means, and a z-test used to assess the difference in proportions. Simple and multiple logistic regression analysis will be carried out between outcome variables (binary) and other variables identified as potential confounders to test for the magnitude and strengths of associations. All tests will be two-tailed and statistical significance set at 0.05.


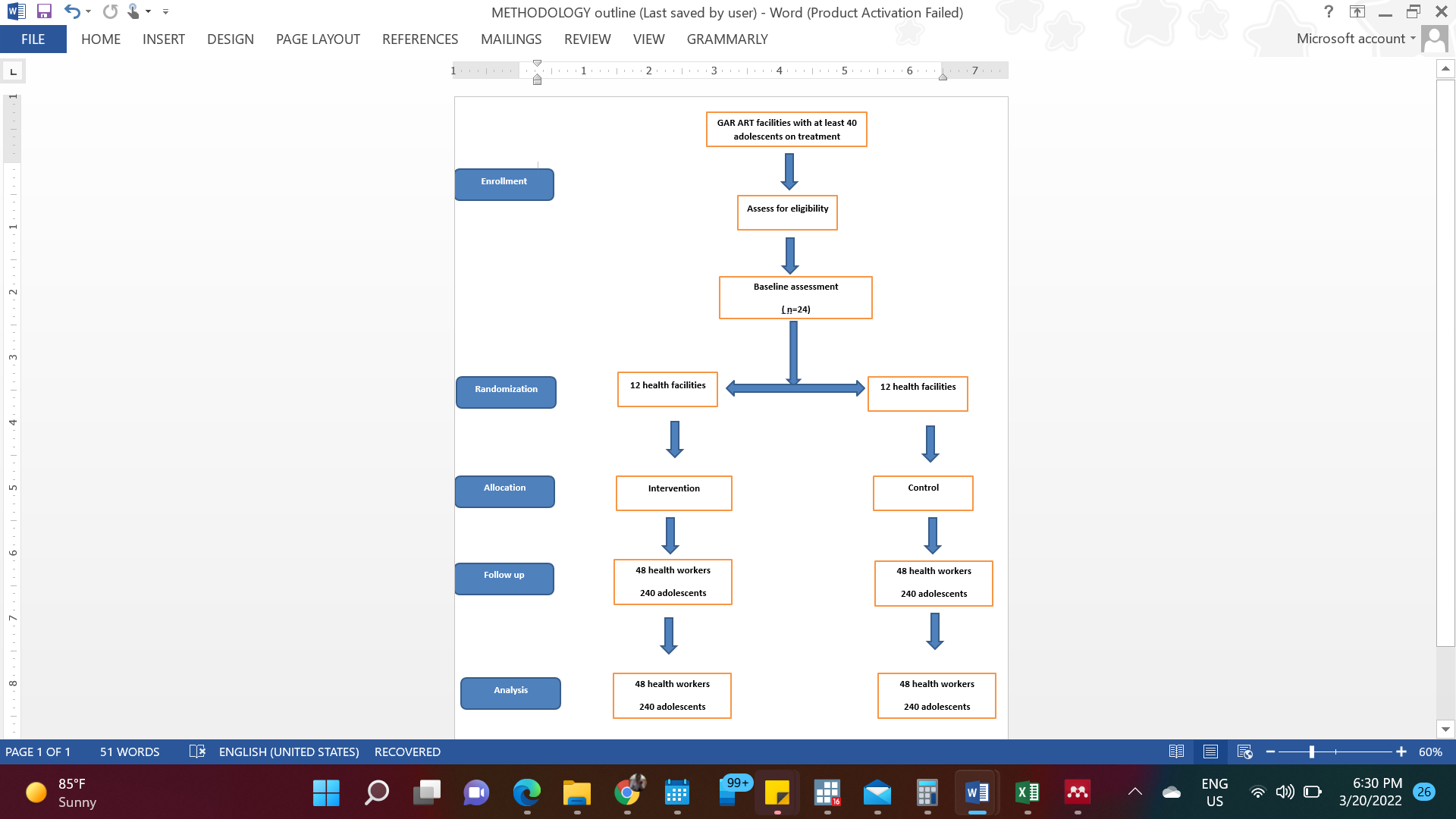


**Figure 5 Flow chart of Cluster randomized Study Procedures**

## **3.5 Expected Outcome**

The study will provide data on the frequency of BP screening among adolescents living with HIV in the selected facilities. The evidence of the effectiveness of the proposed theory of planned behaviour-based intervention on improving healthcare worker adherence to the guidelines and the physiologic measures of the adolescents will also be provided.

## **3.6 Ethical Consideration**

Ethical approval will be sought from the Ghana Health Service Ethics Review Committee. Permission will be sought from the regional and respective municipal health directorates of the study areas and the managers of the health facilities. Permission will also be obtained from the heads of medical records in the respective facilities before extracting data from the client care booklets.

## **3.6.1 Voluntary Participation**

All potential study participants will be made aware that participation in the study is entirely voluntary and that they are free to drop out of the study (and can choose not to disclose reasons for dropping out) at any time with no negative consequence. All participants will sign a written informed consent before enrolment into the study. The consent form will describe the aim of the study, procedures, risks, benefits and compensation. The investigator will be available to answer all questions and allow time for potential participants to think through them and make a decision. Consent from the caregivers of adolescents below 18 years will be sought, followed by participant ascent before participating in the study.

## **3.6.2 Privacy and Confidentiality**

Electronic data will be stored in a password-protected file and hard data in locked cabinets which will only be accessible to the principal investigator and supervisors. Study codes will be assigned to each participant and used as a form of identification on the questionnaires. Data will only be used for academic or publication purposes. The data will be destroyed after ten years.

## **3.6.3 Risks**

There are no foreseen severe physical risks or side effects to the participants due to participating in the study. Participants will be informed to anticipate some minor discomfort while checking both office and ambulatory blood pressures. Nose masks and hand sanitizers will be provided for the study team and participants to minimize the risk of a COVID-19 infection.

## **3.6.4 Compensation**

Adolescent participants coming for follow up assessments outside their scheduled facility visits will have their transportation reimbursed.

## **3.6.5 Declaration of Conflict Of Interest**

The PI has no conflict of interest to declare.

## **3.7 Funding**

The study will be supported by the University of Ghana and the Yale University Academic Partnership for HIV Comorbidity Research Training in Ghana and funded by Fogarty International Center Grant.

## **3.8 Protocol Amendments**

The Ghana Health Service Ethics Review Committee will be informed of all amendments following changes/additions to the protocol and consent form.

**Table 2** **Study Timelines**

| **Timelines** | **2022** | | | | | | | | | | | | | **2023** | | | | |
| --- | --- | --- | --- | --- | --- | --- | --- | --- | --- | --- | --- | --- | --- | --- | --- | --- | --- | --- |
| **Activity** | J | F | M | A | M | J | J | Au | S | O | N | D | J | | F | M | A |  |
| Protocol development |  |  |  |  |  |  |  |  |  |  |  |  |  | |  |  |  |  |
| Ethical approval and stakeholder engagement |  |  |  |  |  |  |  |  |  |  |  |  |  | |  |  |  |  |
| Orientation for research assistants |  |  |  |  |  |  |  |  |  |  |  |  |  | |  |  |  |  |
| Orientation for facility research coordinators |  |  |  |  |  |  |  |  |  |  |  |  |  | |  |  |  |  |
| Baseline assessment |  |  |  |  |  |  |  |  |  |  |  |  |  | |  |  |  |  |
| Orientation on CPG for screening and management of hypertension among adolescents |  |  |  |  |  |  |  |  |  |  |  |  |  | |  |  |  |  |
| Orientation on hypertension among adolescents living with HIV |  |  |  |  |  |  |  |  |  |  |  |  |  | |  |  |  |  |
| Intervention implementation |  |  |  |  |  |  |  |  |  |  |  |  |  | |  |  |  |  |
| Data collection and analysis |  |  |  |  |  |  |  |  |  |  |  |  |  | |  |  |  |  |
| Report writing, presentation and publication |  |  |  |  |  |  |  |  |  |  |  |  |  | |  |  |  |  |

| **Item** | **Budget lines** | **Unit Cost** | **Quantity** | **Duration (days/Months)** | **Frequency** | **Total** | **Comments** |
| --- | --- | --- | --- | --- | --- | --- | --- |
| Ethical Approval and stakeholder engagements | Cost for protocol review | 500 | 1 | 1 | 1 | 500 |  |
|  | Transport to health facilities | 50 | 12 | 1 | 1 | 600 | 2 facilities to be visited per day |
|  | **Subtotal** |  |  |  |  | **1,100** |  |
| Orientation for Research assistants(5) | Snack and Lunch | 60 | 5 | 2 | 1 | 600 | five research assistants to help with data collection. |
|  | Facilitation | 350 | 2 | 2 | 1 | 1,400 |  |
|  | transport for participants | 50 | 5 | 2 | 1 | 500 |  |
|  | Transport for facilitator | 50 | 2 | 2 | 1 | 200 |  |
|  | **Subtotal** |  |  |  |  | **2,700** |  |
| Orientation for Facility Research coordinators | Snack and Lunch | 60 | 24 | 1 | 1 | 1,440 | Each facility will have one study coordinator |
|  | Facilitation | 350 | 2 | 1 | 1 | 700 |  |
|  | Transport for participants | 50 | 24 | 1 | 1 | 1,200 |  |
|  | Transport for facilitator | 50 | 2 | 1 | 1 | 100 |  |
|  | **Subtotal** |  |  |  |  | **3,440** |  |
| Records review | Programming for Kobokolect | 3000 | 1 | 1 | 1 | 3,000 | Data collection to be done on the kobokolect platform |
|  | Transport for data collectors | 50 | 5 | 5 | 2 | 2,500 | a total of about 480 records to be reviewed. The assumption is for 20 records to be reviewed per day per data collector at baseline and 6months after intervention |
|  | Hiring of tablets | 15 | 5 | 9 | 2 | 1,350 |  |
|  | **Subtotal** |  |  |  |  | **6,850** |  |
| orientation on CPG for screening and management of hypertension among adolescents | TPB based questionnaire and pre/post-test papers | 2 | 96 | 1 | 2 | 384 | 96 HCWs(4 per facility) |
|  | Snack and Lunch | 60 | 98 | 2 | 1 | 11,760 |  |
|  | transport for participants | 50 | 96 | 2 | 1 | 9,600 |  |
|  | Transport for facilitator | 50 | 2 | 2 | 1 | 200 |  |
|  | **Subtotal** |  |  |  |  | **21,944** |  |
| Orientation on hypertension among adolescents living with HIV | Snack and Lunch | 60 | 50 | 1 | 1 | 3,000 |  |
|  | transport for participants | 50 | 48 | 1 | 1 | 2,400 |  |
|  | Transport for facilitator | 50 | 2 | 1 | 1 | 100 |  |
|  | **Subtotal** |  |  |  |  | **5,500** |  |
| Intervention package | Paediatric sphygmomanometers | 200 | 12 | 1 | 1 | 2,400 | 10 intervention and 10 control facilities will be used. Controls will receive the package after the follow-up period |
|  | Guidelines for hypertension management | 20 | 12 | 1 | 1 | 240 |  |
|  | Posters | 5 | 12 | 1 | 1 | 60 |  |
|  | BP reference guide | 15 | 12 | 1 | 1 | 180 |  |
|  | **Subtotal** |  |  |  |  | **2,880** |  |
| Follow up | Ambulatory BP machine | 900 | 12 | 1 | 1 | 10,800 |  |
|  | Weighing Scale | 50 | 5 | 1 | 1 | 250 |  |
|  | Stadiometer | 100 | 5 | 1 | 1 | 500 |  |
|  | T&T for adolescent participants | 20 | 480 | 1 | 2 | 19,200 |  |
|  | **Subtotal** |  |  |  |  | **30,750** |  |
|  | **Grand Total** |  |  |  |  | **75,164** |  |

# **4.0 References**

Abbafati, C., Abbas, K. M., Abbasi-Kangevari, M., Abd-Allah, F., Abdelalim, A., Abdollahi, M., Abdollahpour, I., Abegaz, K. H., Abolhassani, H., Aboyans, V., Abreu, L. G., Abrigo, M. R. M., Abualhasan, A., Abu-Raddad, L. J., Abushouk, A. I., Adabi, M., Adekanmbi, V., Adeoye, A. M., Adetokunboh, O. O., … Murray, C. J. L. (2020). Global burden of 87 risk factors in 204 countries and territories, 1990–2019: a systematic analysis for the Global Burden of Disease Study 2019. *The Lancet*, *396*(10258), 1223–1249. https://doi.org/10.1016/S0140-6736(20)30752-2

Adeloye, D., Basquill, C., Aderemi, A. V., Thompson, J. Y., & Obi, F. A. (2015). An estimate of the prevalence of hypertension in Nigeria: A systematic review and meta-analysis. *Journal of Hypertension*, *33*(2), 230–242. https://doi.org/10.1097/HJH.0000000000000413

Agyei-Mensah, S., & Aikins, A. de-G. (2010). Epidemiological Transition and the Double Burden of Disease in Accra, Ghana. *Journal of Urban Health : Bulletin of the New York Academy of Medicine*, *87*(5), 879. https://doi.org/10.1007/S11524-010-9492-Y

Ajzen & Fishbein. (1985). *Theory of Reasoned Action*. https://doi.org/10.4135/9781452276236.n498

Ajzen, I. (1991). The theory of planned behavior. *Organizational Behavior and Human Decision Processes*, *50*(2), 179–211. https://doi.org/10.1016/0749-5978(91)90020-T

Ajzen, I., & Driver, B. L. (1991). Prediction of leisure participation from behavioral, normative, and control beliefs: An application of the theory of planned behavior. *Https://Doi.Org/10.1080/01490409109513137*, *13*(3), 185–204. https://doi.org/10.1080/01490409109513137

Alonso, A., Barnes, A. E., Guest, J. L., Shah, A., Shao, I. Y., & Marconi, V. (2019). HIV Infection and Incidence of Cardiovascular Diseases: An Analysis of a Large Healthcare Database. *Journal of the American Heart Association*, *8*(14). https://doi.org/10.1161/JAHA.119.012241

Alwan, A., Armstrong, T., Bettcher, D., Branca, F., Chisholm, D., Ezzati, M., Garfi eld, R., MacLean, D., Mathers, C., Mendis, S., Poznyak, V., Riley, L., Cho Tang, K., Wild, C., Tsouros Agis, B., Alleyne, G., Armada, F., Banatvala, N., Beaglehole, R., … Zheleznyakov Tim France, E. (2011). *Global status report on noncommunicable diseases 2010 2011*.

Andy. (2010). *Standard Treatment Guidelines*. www.ghndp.org

Appiah, L. T., Sarfo, F. S., Huffman, M. D., Nguah, S. B., & Stiles, J. K. (2019). Cardiovascular risk factors among Ghanaian patients with HIV: A cross-sectional study. *Clinical Cardiology*, *42*(12), 1195–1201. https://doi.org/10.1002/CLC.23273

Arroll, B., Jenkins, S., North, D., & Kearns, R. (1995). Management of hypertension and the core services guidelines: results from interviews with 100 Auckland general practitioners. *The New Zealand Medical Journal*, *108*(994), 55–57. https://pubmed.ncbi.nlm.nih.gov/7885648/

Atibila, F., Hoor, G. ten, Donkoh, E. T., Wahab, A. I., & Kok, G. (2021). Prevalence of hypertension in Ghanaian society: a systematic review, meta-analysis, and GRADE assessment. *Systematic Reviews 2021 10:1*, *10*(1), 1–15. https://doi.org/10.1186/S13643-021-01770-X

Ayoola, O. O., Omotade, O. O., Gemmell, I., Clayton, P. E., & Cruickshank, J. K. (2014). The Impact of Malaria in Pregnancy on Changes in Blood Pressure in Children During Their First Year of Life. *Hypertension*, *63*(1), 167–172. https://doi.org/10.1161/HYPERTENSIONAHA.113.02238

Bae, S., Kim, S. R., Kim, M.-N., Shim, W. J., & Park, S.-M. (2021). Impact of cardiovascular disease and risk factors on fatal outcomes in patients with COVID-19 according to age: a systematic review and meta-analysis. *Heart*, *107*(5), 373–380. https://doi.org/10.1136/HEARTJNL-2020-317901

Bandura, A. (1986). *Social foundations of thought and action: A social cognitive theory. - PsycNET*. Prentice-Hall Series in Social Learning Theory. https://psycnet.apa.org/record/1985-98423-000

Bansal, M. (2020). Cardiovascular disease and COVID-19. *Diabetes and Metabolic Syndrome: Clinical Research and Reviews*, *14*(3), 247–250. https://doi.org/10.1016/j.dsx.2020.03.013

Bao, W., Threefoot, S. A., Srinivasan, S. R., & Berenson, G. S. (1995a). Essential hypertension predicted by tracking of elevated blood pressure from childhood to adulthood: The Bogalusa heart study. *American Journal of Hypertension*, *8*(7), 657–665. https://doi.org/10.1016/0895-7061(95)00116-7

Bao, W., Threefoot, S. A., Srinivasan, S. R., & Berenson, G. S. (1995b). Essential Hypertension Predicted by Tracking of Elevated Blood Pressure From Childhood to Adulthood: The Bogalusa Heart Study. *American Journal of Hypertension*, *8*(7), 657–665. https://doi.org/10.1016/0895-7061(95)00116-7

Bax, J. J., Prendergast, B., & Leclercq, C. (2020). Cardiovascular disease in the COVID-19 pandemic: risk and risk reduction. *European Heart Journal Supplements*, *22*(Supplement_P), P1–P3. https://doi.org/10.1093/EURHEARTJ/SUAA188

Bedu-Addo, G., Alicke, M., Boakye-Appiah, J. K., Abdul-Jalil, I., Giet, M. van der, Schulze, M. B., Mockenhaupt, F. P., & Danquah, I. (2017). In utero exposure to malaria is associated with metabolic traits in adolescence: The Agogo 2000 birth cohort study. *Journal of Infection*, *75*(5), 455–463. https://doi.org/10.1016/J.JINF.2017.08.010

BeLue, R., Okoror, T. A., Iwelunmor, J., Taylor, K. D., Degboe, A. N., Agyemang, C., & Ogedegbe, G. (2009). An overview of cardiovascular risk factor burden in sub-Saharan African countries: A socio-cultural perspective. In *Globalization and Health* (Vol. 5). Global Health. https://doi.org/10.1186/1744-8603-5-10

Benzekri, N. A., Seydi, M., Doye, I. N., Toure, M., Sy, M. P., Kiviat, N. B., Sow, P. S., Gottlieb, G. S., & Hawes, S. E. (2018). Increasing prevalence of hypertension among HIV-positive and negative adults in Senegal, West Africa, 1994-2015. *PLOS ONE*, *13*(12), e0208635. https://doi.org/10.1371/JOURNAL.PONE.0208635

Bigna, J. J., Ndoadoumgue, A. L., Nansseu, J. R., Tochie, J. N., Nyaga, U. F., Nkeck, J. R., Foka, A. J., Kaze, A. D., & Noubiap, J. J. (2020). Global burden of hypertension among people living with HIV in the era of increased life expectancy: A systematic review and meta-analysis. *Journal of Hypertension*, *38*(9), 1659–1668. https://doi.org/10.1097/HJH.0000000000002446

Bobo Kovač, V., & Rise, J. (2011). Predicting the intention to quit smoking in a Norwegian sample. *Nordic Psychology*, *63*(3), 68–82. https://doi.org/10.1027/1901-2276/A000040

Boneparth, A., & Flynn, J. (2009). Evaluation and treatment of hypertension in general pediatric practice. *Clinical Pediatrics*, *48*(1), 44–49. https://doi.org/10.1177/0009922808321677

Bosu, W. K., & Bosu, D. K. (2021a). Prevalence, awareness and control of hypertension in Ghana: A systematic review and meta-analysis. *PLOS ONE*, *16*(3), e0248137. https://doi.org/10.1371/JOURNAL.PONE.0248137

Bosu, W. K., & Bosu, D. K. (2021b). Prevalence, awareness and control of hypertension in Ghana: A systematic review and meta-analysis. *PLOS ONE*, *16*(3), e0248137. https://doi.org/10.1371/JOURNAL.PONE.0248137

Brady, T. M., Solomon, B. S., Neu, A. M., Siberry, G. K., & Parekh, R. S. (2010). Patient-, provider-, and clinic-level predictors of unrecognized elevated blood pressure in children. *Pediatrics*, *125*(6). https://doi.org/10.1542/peds.2009-0555

Browman, G. P. (2000). Improving clinical practice guidelines for the 21st century: Attitudinal barriers and not technology are the main challenges. In *International Journal of Technology Assessment in Health Care* (Vol. 16, Issue 4, pp. 959–968). Int J Technol Assess Health Care. https://doi.org/10.1017/S0266462300103034

Browman, George P. (2005). Clinical practice guidelines and healthcare decisions: Credibility gaps and unfulfilled promises? In *Nature Clinical Practice Oncology* (Vol. 2, Issue 10, pp. 480–481). Nat Clin Pract Oncol. https://doi.org/10.1038/ncponc0286

Cabana, M., CS, R., NR, P., AW, W., MH, W., PA, A., & HR, R. (1999). Why don’t physicians follow clinical practice guidelines? A framework for improvement. *JAMA*, *282*(15), 1458–1465. https://doi.org/10.1001/JAMA.282.15.1458

Carlsen, B., Glenton, C., & Pope, C. (2007). Thou shalt versus thou shalt not: A meta-synthesis of GPs’ attitudes to clinical practice guidelines. In *British Journal of General Practice* (Vol. 57, Issue 545, pp. 971–978). Br J Gen Pract. https://doi.org/10.3399/096016407782604820

Carlsen, B., & Norheim, O. F. (2008). “What lies beneath it all?” - An interview study of GPs’ attitudes to the use of guidelines. *BMC Health Services Research*, *8*. https://doi.org/10.1186/1472-6963-8-218

CDC. (2014). *Centers for Disease Control and Prevention: National... - Google Scholar*. https://scholar.google.com/scholar_lookup?title=National+diabetes+statistics+report:+Estimates+of+diabetes+and+its+burden+in+the+United+States,+2014&publication_year=2014&

Chandar, J., & Zilleruelo, G. (2012). Hypertensive crisis in children. In *Pediatric Nephrology* (Vol. 27, Issue 5, pp. 741–751). Pediatr Nephrol. https://doi.org/10.1007/s00467-011-1964-0

Chatterjee, N. A., & Cheng, R. K. (2020). Cardiovascular disease and COVID-19: implications for prevention, surveillance and treatment. *Heart*, *106*(15), 1119–1121. https://doi.org/10.1136/HEARTJNL-2020-317110

Chatterton-Kirchmeier, S., Camacho-Gonzalez, A. F., McCracken, C. E., Chakraborty, R., & Batisky, D. L. (2015). Increased Prevalence of Elevated Blood Pressures in HIV-Infected Children, Adolescents and Young Adults. *Pediatric Infectious Disease Journal*, *34*(6), 610–614. https://doi.org/10.1097/INF.0000000000000695

Chung, M. K., Zidar, D. A., Bristow, M. R., Cameron, S. J., Chan, T., III, C. V. H., Kwon, D. H., Singh, T., Tilton, J. C., Tsai, E. J., Tucker, N. R., Barnard, J., & Loscalzo, J. (2021). COVID-19 and Cardiovascular Disease. *Circulation Research*, *128*, 1214–1236. https://doi.org/10.1161/CIRCRESAHA.121.317997

CONSORT. (2010). *Consort - CONSORT 2010*. http://www.consort-statement.org/consort-2010

Dahm, P., Yeung, L. L., Gallucci, M., Simone, G., & Schünemann, H. J. (2009). How to Use a Clinical Practice Guideline. *Journal of Urology*, *181*(2), 472–479. https://doi.org/10.1016/j.juro.2008.10.041

Dartnell, J. G. A., Allen, B., McGrath, K. M., & Moulds, R. F. W. (1995). Prescriber guidelines improve initiation of anticoagulation. *The Medical Journal of Australia*, *162*(2). https://doi.org/10.5694/J.1326-5377.1995.TB138435.X

Davies, B., Edwards, N., Ploeg, J., & Virani, T. (2008). Insights about the process and impact of implementing nursing guidelines on delivery of care in hospitals and community settings. *BMC Health Services Research*, *8*, 29–29. https://doi.org/10.1186/1472-6963-8-29

Davis, D. A., Thomson, M. A., Oxman, A. D., & Haynes, R. B. (1995). Changing Physician Performance: A Systematic Review of the Effect of Continuing Medical Education Strategies. *JAMA*, *274*(9), 700–705. https://doi.org/10.1001/JAMA.1995.03530090032018

Davis, K., Perez-Guzman, P., Hoyer, A., Brinks, R., Gregg, E., Althoff, K. N., Justice, A. C., Reiss, P., Gregson, S., & Smit, M. (2021). Association between HIV infection and hypertension: a global systematic review and meta-analysis of cross-sectional studies. *BMC Medicine 2021 19:1*, *19*(1), 1–16. https://doi.org/10.1186/S12916-021-01978-7

Davis, & Taylor-Vaisey, A. (1997). Translating guidelines into practice. A systematic review of theoretic concepts, practical experience and research evidence in the adoption of clinical practice guidelines. *CMAJ*, *157*(4), 408–416. /pmc/articles/PMC1227916/?report=abstract

De Jesus, J. M. (2011a). Expert panel on integrated guidelines for cardiovascular health and risk reduction in children and adolescents: Summary report. In *Pediatrics* (Vol. 128, Issue SUPP.5). Pediatrics. https://doi.org/10.1542/peds.2009-2107C

De Jesus, J. M. (2011b). Expert panel on integrated guidelines for cardiovascular health and risk reduction in children and adolescents: Summary report. In *Pediatrics* (Vol. 128, Issue SUPP.5, pp. S213–S256). American Academy of Pediatrics. https://doi.org/10.1542/peds.2009-2107C

Etyang, A. O., Kapesa, S., Odipo, E., Bauni, E., Kyobutungi, C., Abdalla, M., Muntner, P., Musani, S. K., Macharia, A., Williams, T. N., Cruickshank, J. K., Smeeth, L., & Scott, J. A. G. (2019). Effect of Previous Exposure to Malaria on Blood Pressure in Kilifi, Kenya: A Mendelian Randomization Study. *Journal of the American Heart Association*, *8*(6). https://doi.org/10.1161/JAHA.118.011771

Fahme, S. A., Bloomfield, G. S., & Peck, R. (2018). Hypertension in HIV-Infected Adults. *Hypertension*, *72*(1), 44–55. https://doi.org/10.1161/HYPERTENSIONAHA.118.10893

Falkner, B., & Daniels, S. R. (2004). Summary of the fourth report on the diagnosis, evaluation, and treatment of high blood pressure in children and adolescents. In *Hypertension* (Vol. 44, Issue 4, pp. 387–388). https://doi.org/10.1161/01.HYP.0000143545.54637.af

Falkner, B., & Lurbe, E. (2020). Primordial prevention of high blood pressure in childhood an opportunity not to be missed. In *Hypertension* (Vol. 75, Issue 5, pp. 1142–1150). Hypertension. https://doi.org/10.1161/HYPERTENSIONAHA.119.14059

Field, M. J., & Lohr, K. N. (Eds.). (1990). *Clinical Practice Guidelines*. https://doi.org/10.17226/1626

Fiseha, T., Belete, A. G., Dereje, H., & Dires, A. (2019). Hypertension in HIV-Infected Patients Receiving Antiretroviral Therapy in Northeast Ethiopia. *International Journal of Hypertension*, *2019*. https://doi.org/10.1155/2019/4103604

Fishbein, M. (2012). The Role of Theory in Hiv Prevention. In *The Health Psychology Reader* (pp. 120–126). SAGE Publications Ltd. https://doi.org/10.4135/9781446221129.n8

Flynn, J. T., Kaelber, D. C., Baker-Smith, C. M., Blowey, D., Carroll, A. E., Daniels, S. R., De Ferranti, S. D., Dionne, J. M., Falkner, B., Flinn, S. K., Gidding, S. S., Goodwin, C., Leu, M. G., Powers, M. E., Rea, C., Samuels, J., Simase, M., Thaker, V. V., Urbina, E. M., … Okechukwu, K. (2017). Clinical practice guideline for screening and management of high blood pressure in children and adolescents. In *Pediatrics* (Vol. 140, Issue 3). Pediatrics. https://doi.org/10.1542/peds.2017-1904

Francis, J. J., Eccles, M. P., Johnston, M., Walker, A., Grimshaw, J., Foy, R., Kaner, E. F. S., Smith, L., Bonetti, D., Francis, J., Eccles, M., & Kaner, E. (2004). Constructing questionnaires based on the theory of planned behaviour: A manual for health services researchers. *City University of London*.

Fredric, D., Greenberg, J. H., Parikh, C. R., Devarajan, P., Chui, H., Cockovski, V., Pizzi, M., Palijan, A., Hessey, E., Jia, Y., Thiessen-Philbrook, H. R., & Zappitelli, M. (2021). 24-hour ambulatory blood pressure monitoring 9 years after pediatric cardiac surgery: a pilot and feasibility study. *Pediatric Nephrology*, *36*(6), 1533–1541. https://doi.org/10.1007/S00467-020-04847-2

Gagnon, M.-P., Cassista, J., Payne-Gagnon, J., & Martel, B. (2015). Applying the Theory of Planned Behaviour to understand nurse intention to follow recommendations related to a preventive clinical practice: *Http://Dx.Doi.Org/10.1177/1744987115611715*, *20*(7), 582–593. https://doi.org/10.1177/1744987115611715

Gebrie, A. (2020). Hypertension among people living with human immunodeficiency virus receiving care at referral hospitals of Northwest Ethiopia: A cross-sectional study. *PLOS ONE*, *15*(8), e0238114. https://doi.org/10.1371/JOURNAL.PONE.0238114

Ghana AIDS Commission. (2021a). Ghana HIV Fact Sheet 2020. *Ghana Aids Commission*. https://www.ghanaids.gov.gh/

Ghana AIDS Commission. (2021b). *National and Sub-National HIV and AIDS Estimates and Projections 2020 Report*.

Gheorghe, A., Griffiths, U., Murphy, A., Legido-Quigley, H., Lamptey, P., & Perel, P. (2018). The economic burden of cardiovascular disease and hypertension in low- and middle-income countries: a systematic review. *BMC Public Health 2018 18:1*, *18*(1), 1–11. https://doi.org/10.1186/S12889-018-5806-X

GHS. (2017). *اStandard treatment guidelines*. *12*(1), 145. www.ghndp.org

GHS. (2018). *The Health Sector In Ghana Facts And Figures, 2018 - Datasets - openAFRICA*.

Godin, G., Bélanger-Gravel, A., Eccles, M., & Grimshaw, J. (2008). Healthcare professionals’ intentions and behaviours: A systematic review of studies based on social cognitive theories. *Implementation Science*, *3*(1). https://doi.org/10.1186/1748-5908-3-36

Hagan, J., Shaw, J., & Duncan, P. (2007). *Bright futures: Guidelines for health supervision of infants, children, and adolescents*. https://ebooks.aappublications.org/content/bright-futures-3rd-edition?sso=1&sso_redirect_count=1&nfstatus=401&nftoken=00000000-0000-0000-0000-000000000000&nfstatusdescription=ERROR%3A+No+local+token

Hansen, M. L., Gunn, P. W., & Kaelber, D. C. (2007). Underdiagnosis of hypertension in children and adolescents. *Journal of the American Medical Association*, *298*(8), 874–879. https://doi.org/10.1001/JAMA.298.8.874

Harrison, M. B., Légaré, F., Graham, I. D., & Fervers, B. (2010). Adapting clinical practice guidelines to local context and assessing barriers to their use. *CMAJ*, *182*(2), E78–E84. https://doi.org/10.1503/CMAJ.081232

Horvath, A. R., Kis, E., & Dobos, E. (2010). Guidelines for the use of biomarkers: Principles, processes and practical considerations. In *Scandinavian Journal of Clinical and Laboratory Investigation* (Vol. 70, Issue SUPPL. 242, pp. 109–116). Scand J Clin Lab Invest Suppl. https://doi.org/10.3109/00365513.2010.493424

Hsue, P. Y., & Waters, D. D. (2018). Time to Recognize HIV Infection as a Major Cardiovascular Risk Factor. *Circulation*, *138*(11), 1113–1115. https://doi.org/10.1161/CIRCULATIONAHA.118.036211

Icek Ajzen, M. F. (1980). *Understanding attitudes and predicting social behavior*. https://umbrella.lib.umb.edu/discovery/fulldisplay?vid=01MA_UMB:01MA_UMB&tab=everything&docid=alma99464673503746&context=L&search_scope=MyInst_and_CI&lang=en

IHME. (2019). *GBD Results Tool | GHDx*. Institue for Health Metrics and Evaluation.

Jeffery, R. A., To, M. J., Hayduk-Costa, G., Cameron, A., Taylor, C., Van Zoost, C., & Hayden, J. A. (2015). Interventions to improve adherence to cardiovascular disease guidelines: a systematic review. *BMC Family Practice*, *16*(1), 1–15. https://doi.org/10.1186/S12875-015-0341-7/TABLES/2

Johnson, C. C., & Martin, M. (1996). Effectiveness of a physician education program in reducing consumption of hospital resources in elective total hip replacement. *Southern Medical Journal*, *89*(3), 282–289. https://doi.org/10.1097/00007611-199603000-00005

Karuza, J., Calkins, E., Feather, J., Hershey, C. O., Katz, L., & Majeroni, B. (1995). Enhancing Physician Adoption of Practice Guidelines: Dissemination of Influenza Vaccination Guideline Using a Small-Group Consensus Process. *Archives of Internal Medicine*, *155*(6), 625–632. https://doi.org/10.1001/ARCHINTE.1995.00430060089011

Kelly, R. K., Thomson, R., Smith, K. J., Dwyer, T., Venn, A., & Magnussen, C. G. (2015). Factors Affecting Tracking of Blood Pressure from Childhood to Adulthood: The Childhood Determinants of Adult Health Study. *The Journal of Pediatrics*, *167*(6), 1422-1428.e2. https://doi.org/10.1016/J.JPEDS.2015.07.055

Ko, N. Y., Yeh, S. H., Tsay, S. L., Ma, H. J., Chen, C. H., Pan, S. M., Feng, M. C., Chiang, M. C., Lee, Y. W., Chang, L. H., & Jang, J. F. (2011). Intention to comply with post-exposure management among nurses exposed to blood and body fluids in Taiwan: application of the theory of planned behaviour. *Journal of Hospital Infection*, *77*(4), 321–326. https://doi.org/10.1016/J.JHIN.2010.09.025

Kortteisto, T., Kaila, M., Komulainen, J., Mäntyranta, T., & Rissanen, P. (2010). Healthcare professionals’ intentions to use clinical guidelines: a survey using the theory of planned behaviour. *Implementation Science 2010 5:1*, *5*(1), 1–10. https://doi.org/10.1186/1748-5908-5-51

Kotchen, T. A. (2011). Historical trends and milestones in hypertension research: A model of the process of translational research. In *Hypertension* (Vol. 58, Issue 4, pp. 522–538). Hypertension. https://doi.org/10.1161/HYPERTENSIONAHA.111.177766

Kwarisiima, D., Balzer, L., Heller, D., Kotwani, P., Chamie, G., Clark, T., Ayieko, J., Mwangwa, F., Jain, V., Byonanebye, D., Petersen, M., Havlir, D., & Kamya, M. R. (2016). Population-based assessment of hypertension epidemiology and risk factors among HIV-positive and general populations in rural Uganda. *PLoS ONE*, *11*(5). https://doi.org/10.1371/journal.pone.0156309

Lauer, R. M., Clarke, W. R., Mahoney, L. T., & Witt, J. (1993). Childhood predictors for high adult blood pressure: The Muscatine study. *Pediatric Clinics of North America*, *40*(1), 23–40. https://doi.org/10.1016/S0031-3955(16)38478-4

Leiba, A., Twig, G., Levine, H., Goldberger, N., Afek, A., Shamiss, A., Derazne, E., Tzur, D., Haklai, Z., & Kark, J. D. (2015). Hypertension in late adolescence and cardiovascular mortality in midlife: a cohort study of 2.3 million 16- to 19-year-old examinees. *Pediatric Nephrology 2015 31:3*, *31*(3), 485–492. https://doi.org/10.1007/S00467-015-3240-1

Lewington, S., Clarke, R., Qizilbash, N., Peto, R., & Collins, R. (2002). Age-specific relevance of usual blood pressure to vascular mortality: a meta-analysis of individual data for one million adults in 61 prospective studies. *The Lancet*, *360*(9349), 1903–1913. https://doi.org/10.1016/S0140-6736(02)11911-8

Liu, M. B. (2014). Cardiovascular diseases. In *Chinese Medical Journal* (Vol. 127, pp. 6–7).

Lloyd-Jones, D. M., Hong, Y., Labarthe, D., Mozaffarian, D., Appel, L. J., Van Horn, L., Greenlund, K., Daniels, S., Nichol, G., Tomaselli, G. F., Arnett, D. K., Fonarow, G. C., Ho, P. M., Lauer, M. S., Masoudi, F. A., Robertson, R. M., Roger, V., Schwamm, L. H., Sorlie, P., … Rosamond, W. D. (2010). Defining and setting national goals for cardiovascular health promotion and disease reduction: The american heart association’s strategic impact goal through 2020 and beyond. In *Circulation* (Vol. 121, Issue 4, pp. 586–613). Lippincott Williams & Wilkins. https://doi.org/10.1161/CIRCULATIONAHA.109.192703

Lomas, J., Anderson, G. M., Domnick-Pierre, K., Vayda, E., Enkin, M. W., & Hannah, W. J. (1989). Do practice guidelines guide practice? The effect of a consensus statement on the practice of physicians. *The New England Journal of Medicine*, *321*(19), 1306–1311. https://doi.org/10.1056/NEJM198911093211906

Lurbe, E., Cifkova, R., … J. C.-J. of, & 2009, U. (2019). Management of high blood pressure in children and adolescents: recommendations of the European Society of Hypertension. *Journals.Lww.Com*. https://journals.lww.com/jhypertension/Fulltext/2009/09000/Results_of_blood_pressure_screening_in_a.1.aspx

Mazzuca, S. A., Vinicor, F., Einterz, R. M., Tierney, W. M., Norton, J. A., & Kalasinski, L. A. (1990). Effects of the Clinical Environment on Physicians’ Response to Postgraduate Medical Education. *American Educational Research Journal*, *27*(3), 473–488. https://doi.org/10.3102/00028312027003473

Mbuthia, G. W., Magutah, K., & McGarvey, S. T. (2021). The Prevalence and Associated Factors of Hypertension among HIV Patients. *International Journal of Hypertension*, *2021*. https://doi.org/10.1155/2021/5544916

Mills, K. T., Stefanescu, A., & He, J. (2020). The global epidemiology of hypertension. *Nature Reviews Nephrology 2020 16:4*, *16*(4), 223–237. https://doi.org/10.1038/s41581-019-0244-2

Mulugeta, H., Afenigus, A. D., Haile, D., Amha, H., Kassa, G. M., Wubetu, M., Abebaw, E., & Jara, D. (2021). <p>Incidence and Predictors of Hypertension Among HIV Patients Receiving ART at Public Health Facilities, Northwest Ethiopia: A One-Year Multicenter Prospective Follow-Up Study</p>. *HIV/AIDS - Research and Palliative Care*, *13*, 889–901. https://doi.org/10.2147/HIV.S329838

Nardella, A., Farrell, M., Pechet, L., & Snyder, L. M. (1994). Continuous improvement, quality control, and cost containment in clinical laboratory testing. Enhancement of physicians’ laboratory-ordering practices. *Archives of Pathology & Laboratory Medicine*, *118*(10), 965–968. https://europepmc.org/article/med/7944897

Nartey, E. T. (2021). *Hypertension and Associated Factors in Patients Attending HIV Clinic at the Korle-Bu Teaching Hospital in Accra*. http://ugspace.ug.edu.gh/handle/123456789/32047

NCD Allicance. (2019). *NCDs make up 7 of the world’s top 10 causes of death | NCD Alliance*.

Nelson, J. M., Cook, P. F., & Ingram, J. C. (2014). Utility of the theory of planned behavior to predict nursing staff blood pressure monitoring behaviours. *Journal of Clinical Nursing*, *23*(3–4), 461–470. https://doi.org/10.1111/JOCN.12183

Nerenberg, K. A., Zarnke, K. B., Leung, A. A., Dasgupta, K., Butalia, S., McBrien, K., Harris, K. C., Nakhla, M., Cloutier, L., Gelfer, M., Lamarre-Cliche, M., Milot, A., Bolli, P., Tremblay, G., McLean, D., Padwal, R. S., Tran, K. C., Grover, S., Rabkin, S. W., … Daskalopoulou, S. S. (2018). Hypertension Canada’s 2018 Guidelines for Diagnosis, Risk Assessment, Prevention, and Treatment of Hypertension in Adults and Children. *Canadian Journal of Cardiology*, *34*(5), 506–525. https://doi.org/10.1016/j.cjca.2018.02.022

Nishiga, M., Wang, D. W., Han, Y., Lewis, D. B., & Wu, J. C. (2020). COVID-19 and cardiovascular disease: from basic mechanisms to clinical perspectives. *Nature Reviews Cardiology 2020 17:9*, *17*(9), 543–558. https://doi.org/10.1038/s41569-020-0413-9

Noubiap, J. J. (2020). Hypertension in children in sub-Saharan Africa: primordial prevention is crucial. *PAMJ. 2020; 37:341*, *37*(341), 341. https://doi.org/10.11604/PAMJ.2020.37.341.27387

Noubiap, J. J., Essouma, M., Bigna, J. J., Jingi, A. M., Aminde, L. N., & Nansseu, J. R. (2017). Prevalence of elevated blood pressure in children and adolescents in Africa: a systematic review and meta-analysis. *The Lancet Public Health*, *2*(8), e375–e386. https://doi.org/10.1016/S2468-2667(17)30123-8

Nuamah, K., Affran Bonful, H., Danso Yeboah, J., Antwi Amankwaah, E., Boakye, D., Kwame Owusu, S., Aduako Owusu, A., Amponsah, F., Adomako-Boateng, F., Nang-Beifubah, A., Gyapong, M., Ofosu, A., Garshong, B., & Ansah, E. K. (2017). Characteristics of Inpatient Hypertension Cases and Factors Associated with Admission Outcomes in Ashanti Region, Ghana: An Analytic Cross-Sectional Study. *International Journal of Hypertension*, *2017*. https://doi.org/10.1155/2017/6537956

Oakeshott, P., Kerry, S. M., & Williams, J. E. (1994). Randomized controlled trial of the effect of the Royal College of Radiologists’ guidelines on general practitioners’ referrals for radiographic examination. *The British Journal of General Practice*, *44*(382), 197. /pmc/articles/PMC1238864/?report=abstract

Ogah, O. S., Umuerri, E. M., Adebiyi, A., Orimolade, O. A., Sani, M. U., Ojji, D. B., Mbakwem, A. C., Stewart, S., & Sliwa, K. (2021). SARS-CoV 2 Infection (Covid-19) and Cardiovascular Disease in Africa: Health Care and Socio-Economic Implications. *Global Heart*, *16*(1), 18. https://doi.org/10.5334/GH.829

Oh, J. H., & Hong, Y. M. (2019). Blood pressure trajectories from childhood to adolescence in pediatric hypertension. In *Korean Circulation Journal* (Vol. 49, Issue 3, pp. 223–237). The Korean Society of Cardiology. https://doi.org/10.4070/kcj.2018.0448

Opare, J., Ohuabunwo, C., Agongo, E., Afari, E., Sackey, S., & Wurapa, F. (2013). Improving surveillance for non-communicable diseases in the Eastern Region of Ghana - 2011. *Journal of Public Health and Epidemiology*, *5*(2), 87–94. https://doi.org/10.5897/JPHE12.099

Owolabi, M. O., Sarfo, F., Akinyemi, R., Gebregziabher, M., Akpa, O., Akpalu, A., Wahab, K., Obiako, R., Ovbiagele, B., Sarfo, F. S., Akinyemi, R., Gebregziabher, M., Akpa, O., Akpalu, A., Obiako, R., Ovbiagele, B., Tiwari, H. K., Arnett, D., Lackland, D., … Owolabi, L. (2018). Dominant modifiable risk factors for stroke in Ghana and Nigeria (SIREN): a case-control study. *The Lancet Global Health*, *6*(4), e436–e446. https://doi.org/10.1016/S2214-109X(18)30002-0

Oxman, A. D., Thomson, M. A., Davis, D. A., & Haynes, B. (1995). No magic bullets: a systematic review of 102 trials of interventions to improve professional practice. *CMAJ: Canadian Medical Association Journal*, *153*(10), 1423. /pmc/articles/PMC1487455/?report=abstract

Patel, N. H., Romero, S. K., & Kaelber, D. C. (2012). Evaluation and management of pediatric hypertensive crises: Hypertensive urgency and hypertensive emergencies. *Open Access Emergency Medicine*, *4*, 85–92. https://doi.org/10.2147/OAEM.S32809

Patel, P., Speight, C., Maida, A., Loustalot, F., Giles, D., Phiri, S., Gupta, S., & Raghunathan, P. (2018). Integrating HIV and hypertension management in low-resource settings: Lessons from Malawi. *PLOS Medicine*, *15*(3), e1002523. https://doi.org/10.1371/JOURNAL.PMED.1002523

Peck, R. N., Shedafa, R., Kalluvya, S., Downs, J. A., Todd, J., Suthanthiran, M., Fitzgerald, D. W., & Kataraihya, J. B. (2014). Hypertension, kidney disease, HIV and antiretroviral therapy among Tanzanian adults: a cross-sectional study. *BMC Medicine 2014 12:1*, *12*(1), 1–11. https://doi.org/10.1186/S12916-014-0125-2

Pierre, S., Seo, G., Rivera, V. R., Walsh, K. F., Victor, J. J., Charles, B., Julmiste, G., Dumont, E., Apollon, A., Cadet, M., Saint-Vil, A., Marcelin, A., Severe, P., Lee, M. H., Kingery, J., Koenig, S., Fitzgerald, D., Pape, J., & McNairy, M. L. (2019). Prevalence of hypertension and cardiovascular risk factors among long-term AIDS survivors: A report from the field. *Journal of Clinical Hypertension (Greenwich, Conn.)*, *21*(10), 1558. https://doi.org/10.1111/JCH.13663

Puffer, S., & Rashidian, A. (2004). Practice nurses’ intentions to use clinical guidelines. *Journal of Advanced Nursing*, *47*(5), 500–509. https://doi.org/10.1111/J.1365-2648.2004.03129.X

RHIhub. (2018). *PRECEDE/PROCEED Model - Rural Health Promotion and Disease Prevention Toolkit*. Rural Health Information Hub.

Rico Martín, S., Vassilenko, V., De Nicolás Jiménez, J. M., Rey Sánchez, P., Serrano, A., Martínez Alvarez, M., Calderón García, J. F., & Sánchez Muñoz-Torrero, J. F. (2020). Cardio-ankle vascular index (CAVI) measured by a new device: protocol for a validation study. *BMJ Open*, *10*(10), e038581. https://doi.org/10.1136/BMJOPEN-2020-038581

Rinke, M. L., Singh, H., Brady, T. M., Heo, M., Kairys, S. W., Orringer, K., Dadlez, N. M., & Bundy, D. G. (2019). Cluster Randomized Trial Reducing Missed Elevated Blood Pressure in Pediatric Primary Care: Project RedDE. *Pediatric Quality & Safety*, *4*(5), e187. https://doi.org/10.1097/pq9.0000000000000187

Robinson, M. B. (1994). Evaluation of medical audit. In *Journal of Epidemiology and Community Health* (Vol. 48, Issue 5, pp. 435–440). J Epidemiol Community Health. https://doi.org/10.1136/jech.48.5.435

Rodríguez-Arbolí, E., Mwamelo, K., Kalinjuma, A. V., Furrer, H., Hatz, C., Tanner, M., Battegay, M., Letang, E., & Group, on behalf of the K. S. (2017). Incidence and risk factors for hypertension among HIV patients in rural Tanzania – A prospective cohort study. *PLoS ONE*, *12*(3). https://doi.org/10.1371/JOURNAL.PONE.0172089

Rosenstock, I. M. (1974). The Health Belief Model and Preventive Health Behavior: *Http://Dx.Doi.Org/10.1177/109019817400200405*, *2*(4), 354–386. https://doi.org/10.1177/109019817400200405

Rosser, W. W., & Palmer, W. H. (1993). Dissemination of guidelines on cholesterol. Effect on patterns of practice of general practitioners and family physicians in Ontario. Ontario Task Force on the Use and Provision of Medical Services. *Canadian Family Physician*, *39*, 280. /pmc/articles/PMC2379736/?report=abstract

RTI International. (2016). *Cardiovascular disease costs will exceed $1 trillion by 2035: Nearly half of Americans will develop pre-existing cardiovascular disease conditions, analysis shows*. https://www.sciencedaily.com/releases/2017/02/170214162750.htm

Ryscavage, P., Still, W., Nyemba, V., & Stafford, K. (2019). Prevalence of Systemic Hypertension among HIV-Infected and HIV-Uninfected Young Adults in Baltimore, Maryland. *Southern Medical Journal*, *112*(7), 387–391. https://doi.org/10.14423/SMJ.0000000000001001

Saini, P., Betcherman, L., Radhakrishnan, S., & Etoom, Y. (2021). Paediatric hypertension for the primary care provider: What you need to know. *Paediatrics & Child Health*, *26*(2), 93–98. https://doi.org/10.1093/PCH/PXAA069

Schettler, G., & Brisse, B. (2017). Cardiovascular diseases. *Munchener Medizinische Wochenschrift*, *122*(Suppl. 1), 6–9.

Sekyere, A., & Abena. (2018). *Prevalence of Hypertensive Disorders, Associated Risk Factors and Effect of Specific Organ Function among Adolescents in Some Selected Senior High Schools in Ashanti Region of Ghana*. http://ir.knust.edu.gh:8080/handle/123456789/11049

Shapiro, D. J., Hersh, A. L., Cabana, M. D., Sutherland, S. M., & Patel, A. I. (2012). Hypertension screening during ambulatory pediatric visits in the United States, 2000-2009. *Pediatrics*, *130*(4), 604–610. https://doi.org/10.1542/peds.2011-3888

Shi, S., Qin, M., Shen, B., Cai, Y., Liu, T., Yang, F., Gong, W., Liu, X., Liang, J., Zhao, Q., Huang, H., Yang, B., & Huang, C. (2020). Association of Cardiac Injury with Mortality in Hospitalized Patients with COVID-19 in Wuhan, China. *JAMA Cardiology*, *5*(7), 802–810. https://doi.org/10.1001/jamacardio.2020.0950

Shi, Y., De Groh, M., & Morrison, H. (2012). Increasing blood pressure and its associated factors in Canadian children and adolescents from the Canadian health measures survey. In *BMC Public Health* (Vol. 12, Issue 1). BMC Public Health. https://doi.org/10.1186/1471-2458-12-388

Singh, G. M., Danaei, G., Farzadfar, F., Stevens, G. A., Woodward, M., Wormser, D., Kaptoge, S., Whitlock, G., Qiao, Q., Lewington, S., Di Angelantonio, E., Vander Hoorn, S., Lawes, C. M. M., Ali, M. K., Mozaffarian, D., Ezzati, M., Jørgensen, T., Borch-Johnson, K., Nissinen, A., … Danesh, J. (2013). The age-specific quantitative effects of metabolic risk factors on cardiovascular diseases and diabetes: A pooled analysis. *PLoS ONE*, *8*(7). https://doi.org/10.1371/journal.pone.0065174

Smit, M., Brinkman, K., Geerlings, S., Smit, C., Thyagarajan, K., Sighem, A. van, Wolf, F. de, & Hallett, T. B. (2015). Future challenges for clinical care of an ageing population infected with HIV: a modelling study. *The Lancet Infectious Diseases*, *15*(7), 810–818. https://doi.org/10.1016/S1473-3099(15)00056-0

Song, P., Zhang, Y., Yu, J., Zha, M., Zhu, Y., Rahimi, K., & Rudan, I. (2019). Global Prevalence of Hypertension in Children: A Systematic Review and Meta-analysis. *JAMA Pediatrics*, *173*(12), 1154–1163. https://doi.org/10.1001/JAMAPEDIATRICS.2019.3310

Stein, D. R., & Ferguson, M. A. (2016). Evaluation and treatment of hypertensive crises in children. In *Integrated Blood Pressure Control* (Vol. 9, pp. 49–58). Open Access Emerg Med. https://doi.org/10.2147/IBPC.S50640

Theodore, R. F., Broadbent, J., Nagin, D., Ambler, A., Hogan, S., Ramrakha, S., Cutfield, W., Williams, M. J. A., Harrington, H. L., Moffitt, T. E., Caspi, A., Milne, B., & Poulton, R. (2015). Childhood to Early-Midlife Systolic Blood Pressure Trajectories: Early-Life Predictors, Effect Modifiers, and Adult Cardiovascular Outcomes. *Hypertension*, *66*(6), 1108–1115. https://doi.org/10.1161/HYPERTENSIONAHA.115.05831

Thompson, T. (2014). Theory of Reasoned Action. In *Encyclopedia of Health Communication*. https://doi.org/10.4135/9781483346427.n552

UNAIDS. (2008). Task Shifting- Global Recommendations & Guidelines. In *World Health Organization*.

UNAIDS. (2021a). *Adolescents Living with HIV in Ghana*. https://aidsinfo.unaids.org/

UNAIDS. (2021b). *AIDSinfo*. https://aidsinfo.unaids.org/

UNICEF. (2020). *HIV and AIDS in Adolescents - UNICEF Data*. https://data.unicef.org/topic/adolescents/hiv-aids/

United Nations. (2021). *Education | Department of Economic and Social Affairs*. Sustainable Development. https://sdgs.un.org/goals/goal4%0Ahttps://sdgs.un.org/topics/violence-against-children%0Ahttps://sdgs.un.org/goals/goal16%0Ahttps://sdgs.un.org/topics/education

Webb, T. L., & Sheeran, P. (2006). Does changing behavioral intentions engender behavior change? A meta-analysis of the experimental evidence. *Psychological Bulletin*, *132*(2), 249–268. https://doi.org/10.1037/0033-2909.132.2.249

WHO. (2012). *WHO STEPS Instrument (Core and Expanded) STEPS Instrument Overview* (p. 18). www.who.int/chp/steps

WHO. (2019). *Noncommunicable diseases: Mortality*.

WHO. (2020a). *Differentiated service delivery for chronic disease: Integrating hypertension and diabetes care within DSD for HIV treatment*. https://differentiatedservicedelivery.org/Resources/Resource-Library/Integrating-hypertension-and-diabetes-care-in-DSD

WHO. (2020b). *Global health estimates: Leading causes of death-Ghana*. https://www.who.int/data/gho/data/themes/mortality-and-global-health-estimates/ghe-leading-causes-of-death

WHO. (2021a). *Clinical Guidelines: Antiretroviral Therapy 4.1 Preparing people living with HIV for ART*.

WHO. (2021b). *HIV/AIDS*. https://www.who.int/news-room/fact-sheets/detail/hiv-aids

Wilson, D. M., Taylor, D. W., Gilbert, J. R., Best, J. A., Lindsay, E. A., Willms, D. G., & Singer, J. (1988). A Randomized Trial of a Family Physician Intervention for Smoking Cessation. *JAMA*, *260*(11), 1570–1574. https://doi.org/10.1001/JAMA.1988.03410110078031

World Health Organization. (2020). Global Health Observatory (GHO) data. HIV/AIDS. *World Health Organization*.

World Heart Federation. (2019). *The cost of CVD. Champion Advocates Programme*. World Heart Federation. http://www.championadvocates.org/en/champion-advocates-programme/the-costs-of-cvd

Xu, Y., Chen, X., & Wang, K. (2017). Global prevalence of hypertension among people living with HIV: a systematic review and meta-analysis. *Journal of the American Society of Hypertension*, *11*(8), 530–540. https://doi.org/10.1016/J.JASH.2017.06.004

Yami, A. (2015). *Using the theory of planned behaviour to explore the intentions of a multicultural nursing workforce to comply with policies and procedures in the Prince Sultan Military Medical City (PSMMC)*.

Yang, L., Magnussen, C. G., Yang, L., Bovet, P., & Xi, B. (2020). Elevated Blood Pressure in Childhood or Adolescence and Cardiovascular Outcomes in Adulthood. *Hypertension*, 948–955. https://doi.org/10.1161/HYPERTENSIONAHA.119.14168

Yi, Y., Xu, Y., Jiang, H., & Wang, J. (2021). Cardiovascular Disease and COVID-19: Insight From Cases With Heart Failure. In *Frontiers in Cardiovascular Medicine* (Vol. 8, p. 163). Frontiers. https://doi.org/10.3389/fcvm.2021.629958

Zhou, B., Perel, P., Mensah, G. A., & Ezzati, M. (2021). Global epidemiology, health burden and effective interventions for elevated blood pressure and hypertension. *Nature Reviews Cardiology 2021*, 1–18. https://doi.org/10.1038/s41569-021-00559-8

# **5.0 Appendix**

## **Appendix I: Participant information sheet and consent form(1)**

(For healthcare workers completing open-ended questionnaire for the elicitation study)

**TITLE:** Using a Theory-Based Intervention to Improve Diagnosis and Management of Hypertension among Adolescents Living With HIV in the Greater Accra Region

**PRINCIPAL INVESTIGATOR:** Raphael Adu-Gyamfi

**Background**

Persons living with HIV are at a high risk of developing non-communicable diseases, especially hypertension. Although thought to be an adult disease, hypertension has been found to commence in childhood, with HIV positive children and adolescents found to have higher odds of having elevated blood pressure or being hypertensive. Despite the availability of guidelines for screening and management of hypertension among children and adolescents, there is generally poor surveillance for hypertension amongst them by healthcare workers.

**Purpose of the study**

The purpose of the main study is to examine the use of the Theory of Planned Behaviour (TPB) in predicting the intentions of the healthcare workers who manage HIV clients to comply with guidelines for screening and management of hypertension among children and adolescents. This study involves a self- completion survey using an open-ended questionnaires to elicit responses that will help develop the close ended questionnaire for the main study. The researcher requests your assistance with this study, which aims to help promote the quality of patient care in Ghana.

**Study specific procedures**

If you decide to participate you will answer some questions about your behaviour towards screening and management of hypertension among children and adolescents. It should take approximately 10 minutes to complete the questionnaire.

**Risks and inconveniences**

There are no risks attached to your participation in this study.

**Compensation**

No payment will be made for participation or in compensation for any time lost.

**Confidentiality**

The information we collect about you during the study will be kept private. You will be assigned a code as a means of identification in place of your name. Information gathered will be published in scientific journals, however, your name or identity will not appear in any of the reports or articles we write at the end of the study.

**Ethical consideration**

The study was reviewed by the Ghana Health Service Ethics Review Committee. Permission has also been sought from the Regional Health directorate and the Medical Superintendent/Administrator of your facility.

**Voluntary participation/withdrawal**

Your participation in the study is entirely voluntary. Your decision to participate or not will have no negative consequences. You are at liberty to drop out of the study at any given time (even after you have signed the informed consent form) without reason.

**Provision of information and consent for participants**

If you agree to take part in this study, you will be given a copy of this information sheet to keep. A consent form will be given to you to sign to show that you understand the information that has been given to you and you have willingly agreed to participate in the study.

**Contact persons in case of questions**

If you have any questions concerning your participation in the study feel free to contact the principal investigator, **Raphael Adu-Gyamfi at 0543121078.**

Also, if you think your rights have been harmed as a result of the study or you have other complaints about the study, you can contact the following agency:

Ghana Health Service, Ethics Review Administrator, **Zelma Allotey, 0553893292.**

## **Participant Agreement for Elicitation Study**

The above document describing the benefits, risks and procedures for the research title “**Using a Theory-Based Intervention to Improve Diagnosis and Management of Hypertension among Adolescents Living with HIV in the Greater Accra Region**” has been read and explained to me. I have been given an opportunity to have any questions about the research answered to my satisfaction. I agree to participate as a volunteer.

________________ ________________________________________

Date Name and signature or mark of volunteer

## **Appendix II: Questions for elicitation study**

Your patient is an adolescent (10-17 years) living with HIV. The target behaviour is measuring the patient’s blood pressure (BP). Please take a few minutes to list your thoughts about the following questions:

**Behavioural beliefs (Attitude)**

1. What do you believe are the advantages of measuring the patient’s blood pressure?
2. What do you believe are the disadvantages of measuring the patient’s blood pressure?
3. Is there anything else you associate with measuring the patient’s blood pressure?

**Normative beliefs (Subjective norms)**

1. Are there any individuals or groups who would approve of your measuring the patient’s BP?
2. Are there any individuals or groups who would disapprove of your measuring the patient’s BP?
3. Is there anything else you associate with measuring the patient’s blood pressure?

**Control beliefs (perceived behavioural control)**

1. What factors or circumstances enable you to measure the blood pressure of adolescents living with HIV?
2. What factors or circumstances make it difficult or impossible for you to measure the blood pressure of adolescents living with HIV?
3. Are there any other issues that come to mind when you think about measuring the blood pressure of an adolescent living with HIV?

## **Appendix III: Participant information sheet and consent form (2)**

(For healthcare workers completing theory of planned behavior-based questionnaire)

**TITLE:** Using a Theory-Based Intervention to Improve Diagnosis and Management of Hypertension among Adolescents Living With HIV in the Greater Accra Region

**PRINCIPAL INVESTIGATOR:** Raphael Adu-Gyamfi

**Background**

Persons living with HIV are at a high risk of developing non-communicable diseases, especially hypertension. Although thought to be an adult disease, hypertension has been found to commence in childhood, with HIV positive children and adolescents found to have higher odds of having elevated blood pressure or being hypertensive. Despite the availability of guidelines for screening and management of hypertension among children and adolescents, there is generally poor surveillance for hypertension amongst them by healthcare workers.

**Purpose of the study**

The purpose of this study is to examine the use of the Theory of Planned Behaviour (TPB) in predicting the intentions of the healthcare workers who manage HIV clients to comply with guidelines for screening and management of hypertension among children and adolescents. This study involves a self- completion survey using closed-ended questionnaires in order to ascertain whether healthcare workers’ intentions toward compliance with guidelines are influenced by the TPB constructs. The researcher requests your assistance with this study, which aims to help promote the quality of patient care in Ghana.

**Study specific procedures**

If you decide to participate you will answer some questions about your behaviour towards screening and management of hypertension among children and adolescents. It should take approximately 20 minutes to complete the questionnaire.

**Risks and inconveniences**

There are no risks attached to your participation in this study.

**Compensation**

No payment will be made for participation or in compensation for any time lost.

**Confidentiality**

The information we collect about you during the study will be kept private. You will be assigned a code as a means of identification in place of your name. Information gathered will be published in scientific journals, however, your name or identity will not appear in any of the reports or articles we write at the end of the study.

**Ethical consideration**

The study was reviewed by the Ghana Health Service Ethics Review Committee. Permission has also been sought from the Regional Health directorate and the Medical Superintendent/Administrator of your facility.

**Voluntary participation/withdrawal**

Your participation in the study is entirely voluntary. Your decision to participate or not will have no negative consequences. You are at liberty to drop out of the study at any given time (even after you have signed the informed consent form) without reason.

**Provision of information and consent for participants**

If you agree to take part in this study, you will be given a copy of this information sheet to keep. A consent form will be given to you to sign to show that you understand the information that has been given to you and you have willingly agreed to participate in the study.

**Contact persons in case of questions**

If you have any questions concerning your participation in the study feel free to contact the principal investigator, **Raphael Adu-Gyamfi at 0543121078.**

Also, if you think your rights have been harmed as a result of the study or you have other complaints about the study, you can contact the following agency:

Ghana Health Service, Ethics Review Administrator, **Zelma Allotey, 0553893292.**

## **Participant Agreement for Theory of planned behavior-based questionnaire**

The above document describing the benefits, risks and procedures for the research title “**Using_A_Theory-Based Intervention to Improve Diagnosis and Management of Hypertension among Adolescents Living with HIV in the Greater Accra Region**” has been read and explained to me. I have been given an opportunity to have any questions about the research answered to my satisfaction. I agree to participate as a volunteer.

________________ ________________________________________

Date Name and signature or mark of volunteer

## **Appendix IV: Parental information sheet and consent form(3)**

(For caregivers of adolescents taking part in cluster randomized study)

**TITLE:** Using a Theory-Based Intervention to Improve Diagnosis and Management of Hypertension among Adolescents Living With HIV in the Greater Accra Region

**PRINCIPAL INVESTIGATOR:** Raphael Adu-Gyamfi

**Background**

Persons living with HIV are at a high risk of developing non-communicable diseases, especially hypertension. Although thought to be an adult disease, hypertension has been found to commence in childhood, with HIV positive children and adolescents found to have higher odds of having elevated blood pressure or being hypertensive. Despite the availability of guidelines for screening and management of hypertension among children and adolescents, there is generally poor surveillance for hypertension amongst them by healthcare workers.

**Purpose of the study**

The purpose of this study is to examine the effect of a theory-based intervention on healthcare workers’ adherence to guidelines for screening and management of your ward’s blood pressure during his or her ART clinic visits. The researcher requests you and your ward’s assistance with this study, which aims to help promote the quality of patient care in Ghana.

**Study specific procedures**

If you agree to your ward’s participation, he/she will have her blood pressure checked as recommended. His/her arterial stiffness will also be checked using a procedure that involves placement of blood pressure cuffs on the arms and legs and taking measurements for 15 minutes. The child’s weight and height will also be measured. After the initial assessment, these procedures will be repeated after 6 months.

**Risks and inconveniences**

There are no risks attached to your ward’s participation in this study. He or she might however feel some minor discomfort from the blood pressure cuff placement and inflation.

**Compensation**

No payment will be made for participation or in compensation for any time lost. If the child has to come for an assessment outside their scheduled visit, there will be a GhC 30 transport reimbursement provided.

**Confidentiality**

The information we collect about your ward during the study will be kept private. Your ward will be assigned a code as a means of identification in place of the name. Information gathered will be published in scientific journals, however, your name or identity will not appear in any of the reports or articles we write at the end of the study.

**Ethical consideration**

The study was reviewed by the Ghana Health Service Ethics Review Committee. Permission has also been sought from the Regional Health directorate and the Medical Superintendent/Administrator of your ward’s facility.

**Voluntary participation/withdrawal**

Your ward’s participation in the study is entirely voluntary. Your decision to let your ward participate or not will have no negative consequences. You are at liberty to let your ward drop out of the study at any given time (even after you have signed the informed consent form) without reason.

**Provision of information and consent for participants**

If you agree for your ward to take part in this study, you will be given a copy of this information sheet to keep. A consent form will be given to you to sign to show that you understand the information that has been given to you and you have willingly agreed to participate in the study.

**Contact persons in case of questions**

If you have any questions concerning your ward’s participation in the study feel free to contact the principal investigator, **Raphael Adu-Gyamfi at 0543121078.**

Also, if you think you or your ward’s rights have been harmed as a result of the study or you have other complaints about the study, you can contact the following agency:

Ghana Health Service, Ethics Review Administrator, **Zelma Allotey, 0553893292.**

## **VOLUNTEER AGREEMENT**

The above document describing the benefits, risks and procedures for the research title “**Using_A_Theory-Based Intervention to Improve Diagnosis and Management of Hypertension among Adolescents Living with HIV in the Greater Accra Region**” has been read and explained to me. I have been given an opportunity to have any questions about the research answered to my satisfaction. I agree that my ward should participate as a volunteer.

_______________________ _________________________________________________

Date Name and signature or mark of parent or guardian

**If volunteers cannot read the form themselves, a witness must sign here:**

I was present while the benefits, risks and procedures were read to the child’s parent or guardian. All questions were answered and the child’s parent has agreed that his or her child should take part in the research.

_______________________ _________________________________________________

Date Name and signature of witness

I certify that the nature and purpose, the potential benefits, and possible risks associated with participating in this research have been explained to the above individual.

_______________________ ________________________________________________

Date Name and Signature of Person Who Obtained Consent

## **Appendix V: Adolescent information sheet and assent form (4)**

(For adolescents living with HIV taking part in cluster randomized study)

**TITLE:** Using a Theory-Based Intervention to Improve Diagnosis and Management of Hypertension among Adolescents Living With HIV in the Greater Accra Region

**PRINCIPAL INVESTIGATOR:** Raphael Adu-Gyamfi

**Background**

Persons living with HIV are at a high risk of developing non-communicable diseases, especially hypertension. Although thought to be an adult disease, hypertension has been found to commence in childhood, with HIV positive children and adolescents found to have higher odds of having elevated blood pressure or being hypertensive. Despite the availability of guidelines for screening and management of hypertension among children and adolescents, there is generally poor surveillance for hypertension amongst them by healthcare workers.

**Purpose of the study**

The purpose of this study is to examine the effect of a theory-based intervention on healthcare workers’ adherence to guidelines for screening and management of your ward’s blood pressure during his or her ART clinic visits. The researcher requests you and your ward’s assistance with this study, which aims to help promote the quality of patient care in Ghana.

**Study specific procedures**

If you agree to your ward’s participation, he/she will have her blood pressure checked as recommended. His/her arterial stiffness will also be checked using a procedure that involves placement of blood pressure cuffs on the arms and legs and taking measurements for 15 minutes. The child’s weight and height will also be measured. After the initial assessment, these procedures will be repeated after 6 months.

**Risks and inconveniences**

There are no risks attached to your ward’s participation in this study. He or she might however feel some minor discomfort from the blood pressure cuff placement and inflation.

**Compensation**

No payment will be made for participation or in compensation for any time lost. If the child has to come for an assessment outside their scheduled visit, there will be a GhC 30 transport reimbursement provided.

**Confidentiality**

The information we collect about your ward during the study will be kept private. Your ward will be assigned a code as a means of identification in place of the name. Information gathered will be published in scientific journals, however, your name or identity will not appear in any of the reports or articles we write at the end of the study.

**Ethical consideration**

The study was reviewed by the Ghana Health Service Ethics Review Committee. Permission has also been sought from the Regional Health directorate and the Medical Superintendent/Administrator of your ward’s facility.

**Voluntary participation/withdrawal**

Your ward’s participation in the study is entirely voluntary. Your decision to let your ward participate or not will have no negative consequences. You are at liberty to let your ward drop out of the study at any given time (even after you have signed the informed consent form) without reason.

**Provision of information and consent for participants**

If you agree for your ward to take part in this study, you will be given a copy of this information sheet to keep. A consent form will be given to you to sign to show that you understand the information that has been given to you and you have willingly agreed to participate in the study.

**Contact persons in case of questions**

If you have any questions concerning your ward’s participation in the study feel free to contact the principal investigator, **Raphael Adu-Gyamfi at 0543121078.**

Also, if you think you or your ward’s rights have been harmed as a result of the study or you have other complaints about the study, you can contact the following agency:

Ghana Health Service, Ethics Review Administrator, **Zelma Allotey, 0553893292.**

## **VOLUNTEER AGREEMENT**

By making a mark or thumb printing below, it means that you understand and know the issues concerning this research study. If you do not want to participate in this study, please do not sign this assent form. You and your parents will be given a copy of this form after you have signed it.

This assent form which describes the benefits, risks and procedures for the research titled “**Using_A_Theory-Based Intervention to Improve Diagnosis and Management of Hypertension among Adolescents Living with HIV in the Greater Accra Region**”has been read and or explained to me. I have been given an opportunity to have any questions about the research answered to my satisfaction. I agree to participate.

**Child’s Name:………………………………… Researcher’s Name:……………………………**

**Child’s Mark/Thumbprint………………………..Researcher’s Signature:………………………**

Date:**……………………………………………………** Date: **……………………………………………**

**DATA EXTRACTION FORM**

| **Name of reviewer:……………………………………. Date:………………….** | | | |
| --- | --- | --- | --- |
| **Demographic characteristics** | | | |
| **Question No.** | **Variable** | **Variable code** | **Values** |
| 1) | Folder ID | FID | ………………….. |
| 2) | Study ID | SID | …………………. |
| 3) | District/Municipality | AREA | ………………….. |
| 4) | Facility | FAC | ………………….. |
| 5) | Date of data collection | DATE | dd-mm-yyyy………………………. |
| 6) | Sex | SEX | 0-Female  1-Male |
| 7) | Age (years) | AGE | ……………………………. |
|  |  |  |  |
| 8) | Occupation | OCC | ………………………………. |
| 9) | Highest educational level | EDUC | 1-No formal education  2-Primary school  3-High school  4-Tertiary |
| 10) | Marital status | MSTAT | 1-Single  2-Married  3-co-habitting |
| 11) | Religion | REL | 1-Christian  2-Muslim  3-Other (specify) |
| **Section II – Clinical characteristics** | | | |
| **12** | Date of HIV diagnosis | DD_HIV | dd-mm-yyyy………………………. |
| **13** | Age at HIV diagnosis | **AD_HIV** | ………………….years |
| **14** | HIV Type |  | 1-I 2-II 3-I & II |
| 15 | Weight at baseline | WT_BASE | ……………………………(Kg) |
| 16 | Weight at end line | WT_END | ……………………………(Kg) |
| 17 | Height | HT | ……… (m) |
| 18 | Date of ART initiation | DAI | dd-mm-yyyy………………………. |
| 19 | Age at ART initiation | AAI | …………………………………. |
| 20 | WHO Stage at ART initiation | WSI | 1-1  2-2  3-3  4-4 |
| 21 | Current ART regimen | CAR | ----------------- |
| 21 | Previous ART Regimen | PAR | ……………………….    ………………………. |
| 22 | Viral load at baseline | VL_B | 1-Suppressed  2-Unsuppressed  3-None |
| 23 | Viral load at baseline | VL_B | 1-Suppressed  2-Unsuppressed  3-None |
| 24 | Systolic BP at last visit | SBP | 1………..  2.Not recorded |
| 25 | Diastolic BP at last visit | DBP | 1………..  2.Not recorded |
| 26 | Action taken on BP | BP_ACT | 1. None  2. Rechecked to confirm  3. Given appointment to recheck  4. Counselled on lifestyle  5. Referred |
| 26 | Smoking | SMK | Yes___ No___ Ex____  If Yes: Type___________  Number /day or week_______ |
| 28 | Alcohol use | ALC | Yes___ No___  If Yes (Check one):  Every day___ ≥ Once weekly ___ ≥ Once monthly___ |
