# Supplementary material for: Screening for Hypertension in adolescents living with HIV: Protocol for a cluster randomized trial to improve guideline adherence
Source: PLoS One. 2024 May 3;19(5):e0302016. doi: 10.1371/journal.pone.0302016 (PMC11068165; doi:10.1371/journal.pone.0302016)
Supplement: Attachment — Submitted filename: RESPONSE TO REVIEWERS.docx [file pone.0302016.s003.docx]

School of Public Health,

P.O. BOX 13,

Legon,

Accra.

16-8-2023

THE EDITOR,

PLOS ONE JOURNAL,

Dear Sir,

**RESPONSE TO REVIEWERS**

Kindly find below my response to the reviewer's comments on my protocol manuscript entitled “ Screening for Hypertension in adolescents living with HIV: protocol for a cluster randomized trial to improve guideline adherence.”

**Reviewer 1 Comments and responses**
Abstract
**Comment:** Line 52; ‘them’ refers to what exactly.

**Response:** Please, the “them” refers to Persons Living with HIV(PLHIV) The sentence has been made clearer and now reads as follows: “ Contributing to this is the higher incidence of hypertension among Persons Living with HIV”.

**Introduction**
Comment: Iine 78, seems to indicate available evidence to your study, why doing it.

**Response:** The reason for conducting this study has kindly been added to the introduction section as “Available evidence indicates that multicomponent theory-based guideline implementation strategies can improve adherence to recommendations[21][22,23]. However, there is little data in developing countries, including Ghana, on the implementation of these strategies and their effect on improving healthcare worker adherence to hypertension screening and management guidelines for adolescents. Thus, the study aims to assess the effect of a multicomponent theory of planned behaviour-based intervention on the adherence of antiretroviral therapy clinics to the guidelines for screening and managing hypertension among adolescents living with HIV.”

**Methods**

**Comment 1** : Inclusion criteria must be properly be stated to capture age group above 18 years. What happens to facilities that provide the antiretroviral drugs services? What kind of qualification is accepted?

**Response:** The authors consider these recommendations valuable and the session for study setting, population and participant selection have been revised to capture them.

**Comment 2:** Line 104&105, Why not added to inclusion criteria section.
What is the difference between setting & recruitment and inclusion & exclusion criteria since the contents are similar.

**Response:** Thanks for noting this. The authors have revised the whole section and added them to the inclusion and exclusion criteria

**Comment:** Line 110 to 112, 114 and 115; the language seems more of proposal rather than article for publication.

**Response:** Please the manuscript is a study protocol, which is yet to be conducted

**Comment**: Line 124; how did you come by 10, 80 and 400?

**Response:** Please the section under sample size has been reworded to make this clearer.

**Comment**: What type of blinding was done?

**Response:** The study will be double blinded. This is because, although the participants will be aware of their allocation, the outcome assessors (data collectors) and the study statistician (data analyst) will be kept blind to the allocation of the participants, as described in that section.

**Comment:** Line 166; Yet to be followed or had been followed?

**Response:** Please the study is yet to be conducted so they are yet to be followed.

**Comment:** Line 180 & 189, was the data extracted from client records or questionnaire was used? Needs clarification.

**Response:** Thank you for noting this**.** A data extraction tool will be used to obtain data from client records. The correction has been made and now reads:

“Qualified research assistants will be trained in collecting data by conducting pre-tests of the data extraction tools.”

**Comment:** What becomes of the data after the study?

**Response:** Please the Data Quality Control and Access section of the manuscript has been revised to include this information.

**Comment**: What is new about this study

**Response:** As at the time of manuscript development, this is the first protocol of a study to assess the effect of theory-based interventions on guideline adherence among healthcare workers in Ghana. This has been stated in the discussion section of the manuscript.

**Reviewer 2 Comments and Responses**

**Comment:** Line 103. 28% of the adolescents living with HIV in Ghana receive care in this Region does not seem to be ‘Most’ of them.

**Response:** Thanks for the comment. Please this sentence has been revised and now reads as follows:

“ The study will be conducted in selected antiretroviral therapy facilities in the Public Health sector in the Greater Accra Region, where 28% of the adolescents living with HIV in Ghana receive care”.

**Comment:** Lines 178-185. I assume this will be done at each 20 facilities to achieve the target sample size of 20 x 20 = 400 adolescents. It is unclear from reading the text.

**Response:** Please this comment has been considered and the section under Sample Size has been revised to make this clearer.

**Comment:** Line 192. The fact that the principal investigator will have access to the data is an anomaly. I suggest that especially the PI is kept blind to the data as the knowledge of interim data could affect the conduct of the trial.

**Response:** The authors consider this comment very relevant. The PI’s supervisors will have access to the electronic version of the data and access will only be granted to the PI after the study is completed. The section under Data Quality Control and Access has been revised to reflect this.

**Comment:** Line 198. I would avoid square root transformations as it is cumbersome to transform back to the original scale. Use non-parametric tests on the natural scale instead.

**Response:** The study team has considered the recommendation and agreed that there won't be any data transformation. That statement has therefore been deleted from the data analysis section.

**Comment**: Line 208. If a logistic regression is used it should be Odds Ratios or Risk Ratios, not Rate Ratios.

**Response:** The comment has been considered by the study team. Poisson regression will rather be conducted instead of logistic regression. Prevalence ratios, instead of rate ratios will also be determined. The section under data analysis has thus been revised to reflect this change.
